# What Contribution Could Industrial Symbiosis Make to Mitigating Industrial Greenhouse Gas (GHG) Emissions in Bulk Material Production?

**DOI:** 10.1021/acs.est.2c01753

**Published:** 2022-06-30

**Authors:** Lukas Gast, André Cabrera Serrenho, Julian M. Allwood

**Affiliations:** Department of Engineering, University of Cambridge, Trumpington Street, Cambridge CB2 1PZ, United Kingdom

**Keywords:** industrial symbiosis, GHG emissions mitigation, bulk material production, industrial emissions

## Abstract

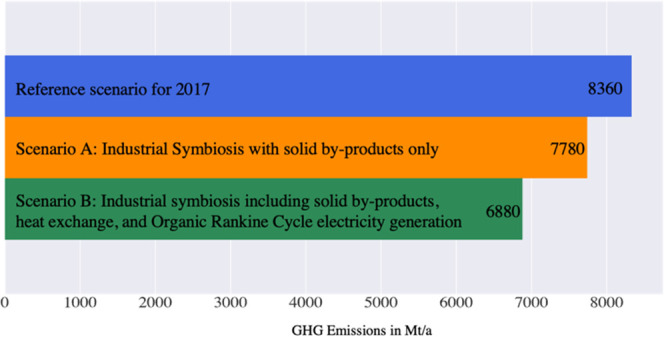

In industrial symbiosis, byproducts and wastes are used to substitute other process inputs, with the goal of reducing the environmental impact of production. Potentially, such symbiosis could reduce greenhouse gas emissions; although there exists literature exploring this at specific industrial sites, there has not yet been a quantitative global assessment of the potential toward climate mitigation by industrial symbiosis in bulk material production of steel, cement, paper, and aluminum. A model based on physical production recipes is developed to estimate global mass flows for production of these materials with increasing levels of symbiosis. The results suggest that even with major changes to byproduct utilization in cement production, the emission reduction potential is low (7% of the total bulk material system emissions) and will decline as coal-fired electricity generation and blast furnace steel production are phased out. Introducing new technologies for heat recovery allows a greater potential reduction in emissions (up to 18%), but the required infrastructure and technologies have not yet been deployed at scale. Therefore, further industrial symbiosis is unlikely to make a significant contribution to GHG emission mitigation in bulk material production.

## Abstract 2

This paper quantifies the potential contribution of industrial symbiosis to industrial GHG emission mitigation and helps to reprioritize emission mitigation strategies.

## 1 Introduction

Global production of materials is increasing, contributing to global greenhouse gas (GHG) emissions and climate change.^1^ The OECD^2^ notes that, with current trends, material demand and production is expected to double by 2060. Similarly, IEA^3^ assumes increasing bulk material production driven by increasing material demand in Asia. Some byproducts from bulk material production processes can be used to substitute other energy- and emission-intensive materials, hence reducing raw material consumption and GHG emissions. In its fifth Assessment Report, the IPCC discusses this substitution as a means to mitigate GHG emissions on a process level, industry park level, and national level via industrial symbiosis (IS).^4^ This concept can be described as the utilization of the output of one production process as an input stream for another production process.^5^ The economic benefits include the selling of waste, the valorization of byproduct flows,^6^ and the avoidance of costs, such as landfill taxes.

Environmental benefits may include resource and emission savings: the literature on industrial symbiosis often uses the example of the production site in Kalundborg^7^ to explain the concept and highlight its environmental benefits. Prominent examples of byproduct exchanges include the use of slags and ashes^8^ or other cementitious materials^9^ in cement production. Other examples include the cascading of cooling water^10^ and the use of heat exchange networks,^11^ which reduce the demand for materials and fuels and hence emissions. The literature on industrial symbiosis has explored and described several industrial waste streams and case studies of industrial symbiosis, e.g., as summarized in the Yale IS case study database^12^ and a recent review by Neves et al.^13^ The database provides a detailed overview of the case studies, but their GHG emission savings have not yet been estimated. Indeed, Liu et al.^14^ commented on the scarcity of literature evaluating the GHG emission saving potential of industrial symbiosis. Recent analyses have tried to assess the potential of an industrial symbiosis on a country level, e.g., a quantitative assessment of energy conservation and emission reduction in steel and cement production in China by Cao et al.^15^ Their analysis estimates that up to 35.7 Mt of coal equivalent could be saved through the utilization of slag and heat recovery options, referred to as industrial symbiosis technologies. Whereas slag utilization is high in some countries, the utilization rates in China are at about 30% and provide the potential for further utilization.^16^ Ramaswami et al.^17^ assessed the opportunities for urban symbiosis in China through heat utilization and estimate a potential reduction of CO_2_ emissions ranging from insignificant (1%) to up to 37% in areas close to industrial clusters, mainly realized through the utilization of low-grade waste heat and replacement of coal and natural gas in these cities. The methodology was applied to other urban clusters, e.g., for 111 Chinese Industrial Parks,^18^ the cluster around Shenyang City,^19^ and the Chinese cluster Yongcheng.^20^

To highlight the benefits of industrial symbiosis, the IPCC^4^ refers to publications that cover case studies including the use of alternative fuels and cementitious materials to substitute for clinker. For example, the report refers to an emission reduction in cement plants of up to 10–20% through substituting fossil fuels with municipal solid waste.^21^ However, their analysis only provides limited evidence for the reduction of actual emissions due to industrial symbiosis. Instead, the fossil-fuel-related emissions are mostly replaced with emissions from combusting these alternative fuels. The second category is the utilization of blast furnace slag and fly ash to replace emission-intensive clinker. Hinkel et al.^22^ highlighted the potential for emission savings of better waste management that mitigates emissions originating from landfilling and open burning of waste. They provide a detailed overview of processing wastes for coincineration and conclude that emission reduction depends on the accounting of biogenic and fossil-based emissions.

The IPCC report recommends industrial symbiosis as a strategy but to what extent can it help to mitigate climate change and what is required to realize this potential in practice? The objective of this paper is to provide a quantitative estimate of the maximum global potential for industrial greenhouse gas emission mitigation by industrial symbiosis in the bulk material industries.

## 2 Modeling the Global GHG Reduction Potential of Industrial Symbiosis

A global model of industrial symbiosis is developed in four steps. First, a mathematical model is developed to simulate industrial production and optimize the utilization of byproducts of a global industrial production system. “Production recipes”, physical input–output coefficients for major production processes, are then obtained for the production processes from a detailed literature review. Third, the model is used to estimate current global mass flows as a reference scenario and a procedure for validation is described. Finally, two scenarios of industrial symbiosis are developed to assess potential emission mitigation.

### 2.1 Definition of the Model of Global Industrial Symbiosis

The model assumes that the world’s industrial bulk material production comprises *P* processes, including material production processes, electricity and steam generation, and heat exchange (between different phases and across different temperature spans). The material processes include primary and secondary routes for all major bulk material production processes and consist of several subprocesses with inputs and outputs. The processes are indexed by *j* (*j* ∈ {1, 2, ..., *P*}), and the rate at which each process operates is *r*_*j*_. Across all industrial activities, there are *N* substances defined by type and temperature band to allow for an optimization of the mass flow and heat exchange across the streams: the process heat is provided through the direct use of fuels and mass flows of hot air and steam for providing the process heat. For solid flows, only the relevant temperature levels (e.g., output temperature of the processes and for which heat exchangers exist) are included. Uniquely, electricity demand is not represented as a mass flow but as energy (kWh/kg_output_). The substances are indexed by *i* (*i* ∈ {1, 2,..., *N*}), so the amount of substance *i* is *x*_*i*_. One of the substances is GHG emissions (kg_CO_2e__/kg_output_), denoted *g*_*j*_ for the emissions released by process *j*.

The system converts inputs provided from the nature (e.g., ores) and other industrial processes (e.g., scrap), ***u***, as well as reused byproducts of the other processes to production outputs ***y***. These split among final demand from consumers ***ŷ*** and wastes ***w***. The coefficients of the production processes in the system are stored in a coefficient matrix ***A***, with entries *a*_*ij*_. When process *j* operates at rate *r*_*j*_, it causes an exchange of substances
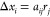
1

Coefficients *a* are known or estimated from the literature. Coefficients *a* are negative when they are inputs to the process and positive when they are outputs. Mass balance of the processes is ensured with ∑_*i*_*a*_*i,j*_ = 0 ∀*j* (noting that this sum excludes the single element describing electricity). When all processes operate together, eq 1 becomes
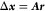
2

GHG emissions ***g*** are the entries in the first row of the matrix. The sum of GHG emissions *g* of all processes can be calculated as
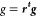
3

Each process requires some inputs (indicated by negative values of *a*) that are either supplied externally or are outputs of other processes. The inputs to the industrial system must either be found in nature or produced by other processes as intermediary products. The byproducts of the production processes can be immediately used in another downstream process if a process for their utilization exists. The output of the industrial system is
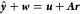
4

The minimization of the system’s GHG emissions *g* is subject to the constraint that the output must meet a global material demand ***ŷ***, which is defined externally. Entries in the vector of the material demand (***ŷ***) are material demand in 2017 for all bulk materials and zero for intermediate and other goods. Process rates ***r*** and system outputs ***y*** must be non-negative. There are restrictions on the supply and availability of (secondary) resources provided by other external processes. Thus, for the external supply, an upper limit ***c*** is applied to some elements of ***u***. The entries of ***c*** will be zero for resources that are not available in nature, and finite otherwise. The production process rates ***r*** and inputs from other processes ***u*** are the decision variables. Minimization with constraints can be expressed as follows
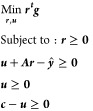
5

### 2.2 Compiling the Production Recipes for Estimating the Input and Output Flows of the Industrial Production Processes

Although process production recipes are implied by life-cycle-inventory (LCI) analysis, harmonizing system boundaries can prove challenging.^23^ Brogaard et al.^24^ showed that GHG emissions across LCI data sets for the same processes vary significantly and also note that the decreasing emission intensities of material production in recent years are not reflected in LCI data sets. Hence, the LCI databases were not used to generate estimates of physical input and output flows. Instead of using LCI databases, spatial upscaling using process-level data is used in this analysis. This approach has similarities to hybrid multiregional input–output (MRIO) approaches that include physical supply and use of materials.^25^ For example, a hybrid MRIO approach was used to combine physical waste flow data with economic data to provide an estimate of the physical waste flows for Australia.^26^

Physical input–output recipes and the final production demand in 2017 are used in this analysis. The compilation of the process recipes includes the following steps: (1) defining the key processes for the production of the major bulk materials, (2) collecting production coefficients of these processes, and (3) adding further auxiliary and symbiosis processes.

#### 2.2.1 Step 1: Defining the Key Processes in the Model

The analysis covers the production of steel, aluminum, paper, and cement, which drives around 55% of global industrial emissions.^4^ The production of chemicals, e.g., ammonia and petrochemicals, was not included since they are produced in integrated production parks and clusters already. Plant-level potentials exist for improving the efficiency of combustion and heat transfer processes as highlighted in a case study of exergy losses.^27^ The production system boundary includes the main process stages for producing the materials as well as electricity and heat generation. For mining, average GHG emissions from mining operations were used as an approximation. Downstream casting and manufacturing processes and direct and indirect emissions from these processes were excluded from this analysis. The production process chains and their boundaries are based on published material flow analyses: steel,^28^ aluminum,^29^ clinker, and cement production^30^ and paper production.^31^

#### 2.2.2 Step 2: Collecting Physical Input and Output Flows of the Processes

Input and output material flows are collected and normalized for all subprocesses in the production routes per unit of output of that subprocess, in MJ or kg_input_/kg_output_. Information on industrial material flows is available in published data from industrial associations (e.g., World Steel Association^32^), best-available technology (BAT) documents from the European Commission (e.g., BAT documents on paper production^33^), case studies in journal articles, and other technical reports. For the analysis, global average data from different years and sources was used. This included data on global average inputs and outputs (e.g., for steel, cement, and aluminum production) and European paper production data.

Full details of each production process including coefficients, sources, and assumptions are provided in the Supporting information (SI). Where available, global survey data and average production coefficients are used. Missing data was found from proxy sources assuming that, where data from different years was used, the proportions of processes remained the same and that geographical variations could be disregarded. While industrial associations (e.g., the World Steel Association) collect some of this information from their members and production sites worldwide, this information is not available for all processes and byproducts. The BAT documents by the European Commission, e.g., on iron and steel production,^34^ include a large number of production sites in the European Union and provide the ranges and average distributions of the coefficients for most processes. However, the information is not provided for all subprocesses but only on an aggregated level. The sources used for the production recipes are summarized in Table 1. This approach was chosen to use the best-available public data on the production processes. The implications for immediate potentials for symbiosis, however, might be different for specific regions that have more (or less) efficient production processes and utilization of byproducts.

**Table 1:** Overview of the Bulk Material Processes and Sources Used for the Production Recipes

sector	explanation of data used	data sources
steel	global average flow data for BF steel production and EAF production from a representative set of steel production sites using data from World Steel Association. Additional information from the best-available technology (BAT) documents by the European Commission was used for missing flows	(32, 34−40)
cement	global survey data (excluding China) collected by the getting numbers right (GNR) initiative and additional information on flows from peer-reviewed case studies and technical reports on the use of cementitious materials	(9, 41−47)
aluminum	data on global aluminum flows from the World Aluminum Association and European Aluminum Association for Europe. Additional information on waste heat recovery potentials from industrial case studies	(48−54)
paper	process-level data for paper production from BAT in the European Union documents, two peer-reviewed analyses of material flows in paper production systems, and (representative) emission data from LCI database ecoinvent	(31, 40, 55−61)

The energy flows within the production recipes are reported in MJ/kg for primary energy and in kWh/kg for electricity. For processes requiring fuel for chemical reactions as well as heat (e.g., in blast furnaces), the fuels were directly added to the mass balances of the production recipes (in kg/kg). If steam is used to provide heat in industrial processes (e.g., paper production), boiler processes for steam generation were introduced to convert fuels into steam at different temperature bands based on the global average heat and electricity demand reported in Worrell et al.^40^ and Rogers et al.^61^ Steam generation from hot flue gases was included in the scenarios of industrial symbiosis using simplified mass and energy balances and using the average efficiency of industrial boilers in Paoli and Cullen.^62^ For steel production, best practice information in the European Commission^63^ and Worrell et al.^40^ was used. Similarly, the best-available technology documents by the European Commission were used for aluminum,^50^ paper,^57^ and cement.^64^ The list of processes in the model, a detailed flow chart, the mass-balanced recipes, and data sources are provided in the SI.

Model uncertainty arises from process simplification, geographical variations, and technology variations within each sector, but the accuracy of the recipes cannot easily be verified. The BAT documents of the European Commission report large ranges for the mass data of the byproduct flows. The reported byproducts per tonne of liquid steel illustrate this: 2–22 kg/t sludge, 150–346 kg/t for blast furnace (BF) slags, 3–18 kg/t for top gas dust.^63^ The process recipes were then checked for mass balance, and a flow for imbalances was calculated and added if the process did not have a mass balance. The flows in the recipes were rounded to the nearest kilogram and all flows smaller than 0.01 kg/kg were removed from the mass balances or aggregated into groups of flows. All flows in the summary of the results were reported to the nearest million tonnes as a precaution.

#### 2.2.3 Step 3: Adding Further Auxiliary and Symbiosis Processes

Global average greenhouse gas emissions reported by IEA^65^ were used for electricity generation. The production recipes of other auxiliary processes were simplified and calculated from the data on average global GHG emissions in IEA.^65^ The combustion processes for black liquor (for heat) and blast furnace gases (for electricity generation) were included with typical stoichiometric combustion coefficients.

Processes for industrial symbiosis were identified by a review of relevant case studies. For example, BF slag can be processed to substitute clinker. Additionally, processes for medium- and high-temperature heat recovery were included, e.g., for utilizing the clinker cooling flue gases as reported in Karellas et al.^45^ as well as Fellaou and Bounahmidi.^46^ Technologies for electricity generation from medium- and low-temperature waste heat using organic Rankine cycle (ORC) turbines were taken from different case studies. These included electricity generation from the heat in clinker cooler gas,^66^ sinter and blast furnace flue gas,^67^ and aluminum flue gas.^68^ Ramaswami et al.^17^ provide examples of options for low- and medium-temperature heat utilization in district heating systems. The implementation of the heat networks requires additional infrastructure and the distance might limit the economic feasibility, e.g., as highlighted in Santin et al.^69^ Since some production sites might be distant from district heating systems and the model already contains strong assumptions about the distance and utilization, further processes for low- and medium-temperature heat utilization were not included.

### 2.3 Model Validation with Reported Byproduct Flows for 2017

Global material demand (***ŷ***) in 2017 was 1207 Mt blast furnace steel,^70^ 472 Mt electric arc furnace steel,^70^ 64 Mt primary aluminum,^51^ 29 Mt secondary aluminum,^71^ 4050 Mt cement,^72^ and 419 Mt paper,^73^ including 263 Mt produced from virgin pulp.^73^ The model of eq 5 can be solved to predict the other material flows of the production system and compared to other published data for model validation. For that, the estimates of the mass flow in the reference scenario are compared with the reported mass flows to validate the estimates for the main and byproducts. This approach was chosen since no other public database exists yet that could be used to validate the estimates.

Global flows of greenhouse gas emissions and some byproducts are reported by industrial associations. For steel production, the information provided in the production data by World Steel Association^32^ was used. For aluminum, data from World Aluminum^51^ and a report on byproduct management^74^ were used. For cement, the average inputs and outputs from participating countries of the GNR initiative^42^ and the reported global average mix by IEA^65^ were used. For paper production, the global flows reported in van Ewijk et al.^75^ were used.

### 2.4 Definition of the Two Scenarios of Industrial Symbiosis

A comparison of outputs from the reference scenario with these from two scenarios of industrial symbiosis (Scenarios A and B) is used to predict the feasible maximum reduction of GHG emissions. Scenario A assumes global deployment of all available technologies for solid byproduct utilization, in particular related to the exchange of cementitious materials such as fly ash or blast furnace slag. Scenario B augments this with established technologies for heat recovery, including the possible use of organic Rankine cycle generation. All main process recipes are the same across all scenarios.

**Figure 1:**
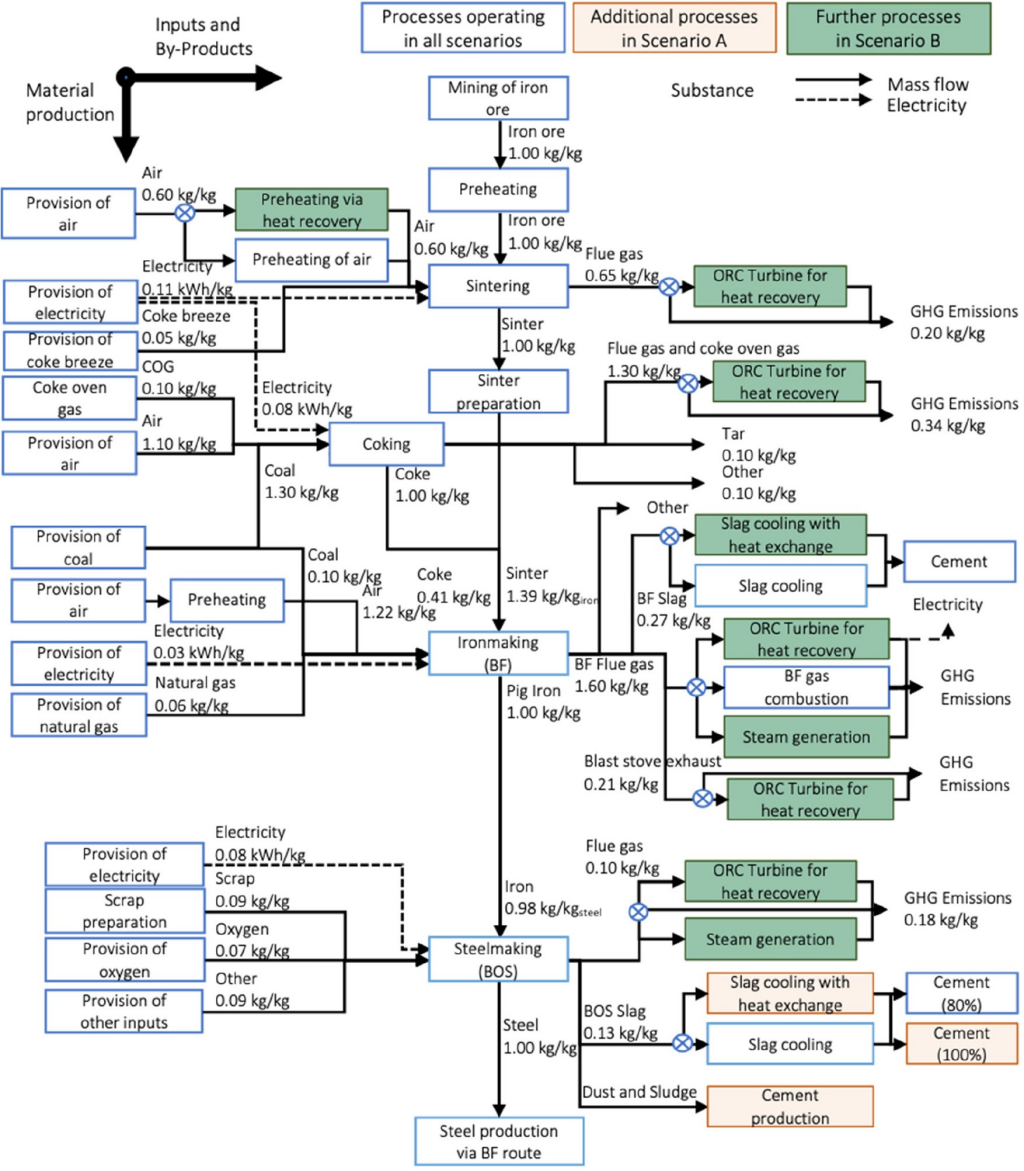
Blast furnace steel production process route with the processes of industrial symbiosis and heat exchange in Scenarios A and B.

A table with the list of the symbiosis processes is provided in the SI. As an example, the production processes and symbiosis processes for steel are summarized in Figure 1. The byproducts, which include high- and medium-temperature flows, can be used in different downstream processes, which operate at rate *r* and are included in Scenarios A and B. For the utilization of waste heat in heat networks, technologies for heat recovery using ORC turbines were added. The analysis does not include the exchanges of and cascading of water, which is included as a byproduct in the general definition of industrial symbiosis.

## 3 Results

**Figure 2:**
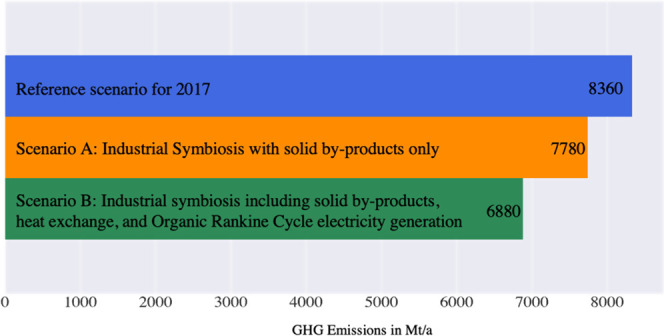
Overall changes in GHG emissions for material production in 2017.

The overall result summarized in Figure 2 is that global production of the four materials causes 8360 Mt_CO_2e__ emissions in the reference scenario. This is reduced by 7% for Scenario A (current symbiosis technologies) and 18% for Scenario B (full symbiosis and electricity generation with ORC turbines).

### 3.1 Estimates of the Inputs and Outputs of the Global Bulk Material Production System

The mass flows in the reference scenario are validated by comparing the estimates of the model with other mass flow analyses. Table 2 compares model predictions of byproduct mass flows with those identified in the literature. All but one of the model predictions are close to the reported flows. The major difference is for black liquor. Van Ewijk et al.^75^ reported a global production of black liquor of 152 Mt in 2012. Even if adjusted for the overall pulp production in 2017 (182 Mt in 2012 to 187 Mt in 2017), this is significantly different from the model estimate. The process recipe used in the model is based on the specific production of black liquor from the pulping process (1.7 t/t pulp in Naqvi et al.^76^ and the BAT from the European Commission^33^). The difference in Table 2 could be because in reality some black liquor is used internally for heat generation and only that leaving the production site was reported by Van Ewijk et al.^75^

**Table 2:** Overview of the Industrial Main Products and Byproducts Estimated in the Model and Estimates of the Scale of Byproducts Reported in the Literature

name of flow	process	year	reported flow (Mt)	model result (Mt)	difference (Mt)	difference (%)	ref
basic oxygen furnace slag	steel	2017	150	150	0	0	(32)
blast furnace slag	steel	2017	330	330	0	0	(32)
BOF dust and sludge	steel	2017	4	4	0	0	(32)
EAF slag	steel	2017	80	80	0	0	(32)
direct CO_2_ emissions	steel	2017	2210	2280	80	3	(32)
GHG emissions	cement	2017	3460	3410	50	–1	(77)
red mud and bauxite residues	aluminum	2015	120	120	0	0	(53)
papermaking waste	paper	2012	20	20	0	0	(75)
paper recycling waste	paper	2012	40	40	0	0	(75)
black liquor	paper	2012	150	360	210	58	(75)

The model estimates that electricity generation from organic Rankine cycle (ORC) turbines could replace up to 140 TWh of electricity in steel, cement, and aluminum production if deployed globally. For steel and cement production in the European Union, Campana et al.^78^ conducted a process-level analysis based on energy audits and find a potential for ORC electricity generation of approximately 2.8–4.6 TWh/year for cement and 3.7–6.0 TWh/year for steel production (representing 0.46 and 0.58% of industrial electricity consumption in the European Union), which corresponds to more than 8 Mt of GHG emissions. A recent assessment of the economic potential for industrial production in Germany by Pili et al.^79^ estimates a recovery potential of 1.3–2.3 TWh/year for steel and 0.7–1.3 TWh/year for cement production using waste heat factors from the literature. Given that steel and cement production in the European Union represent about 10% (steel) and 5% (cement) of global production, and assuming a global scale-up of these technologies, the global potential of ORC technologies using the estimates of these studies could be up to 60 TWh (steel) and 92 TWh (cement), providing additional validation for the model predictions.

A sensitivity analysis was also conducted to assess the robustness of the model with regard to changes in the input parameters (GHG emission coefficients, material demand, and fly ash supply). The results of the sensitivity analysis are provided in the Supporting Information in Section 4. The relative changes of overall emissions due to changes in the process-level emission factors lead to similar increases and decreases in system GHG emissions across the scenarios. The changes in the availability of the secondary materials lead to small changes in the overall emissions in the reference scenario.

### 3.2 Potential Contribution of Industrial Symbiosis to GHG Emission Mitigation

**Figure 3:**
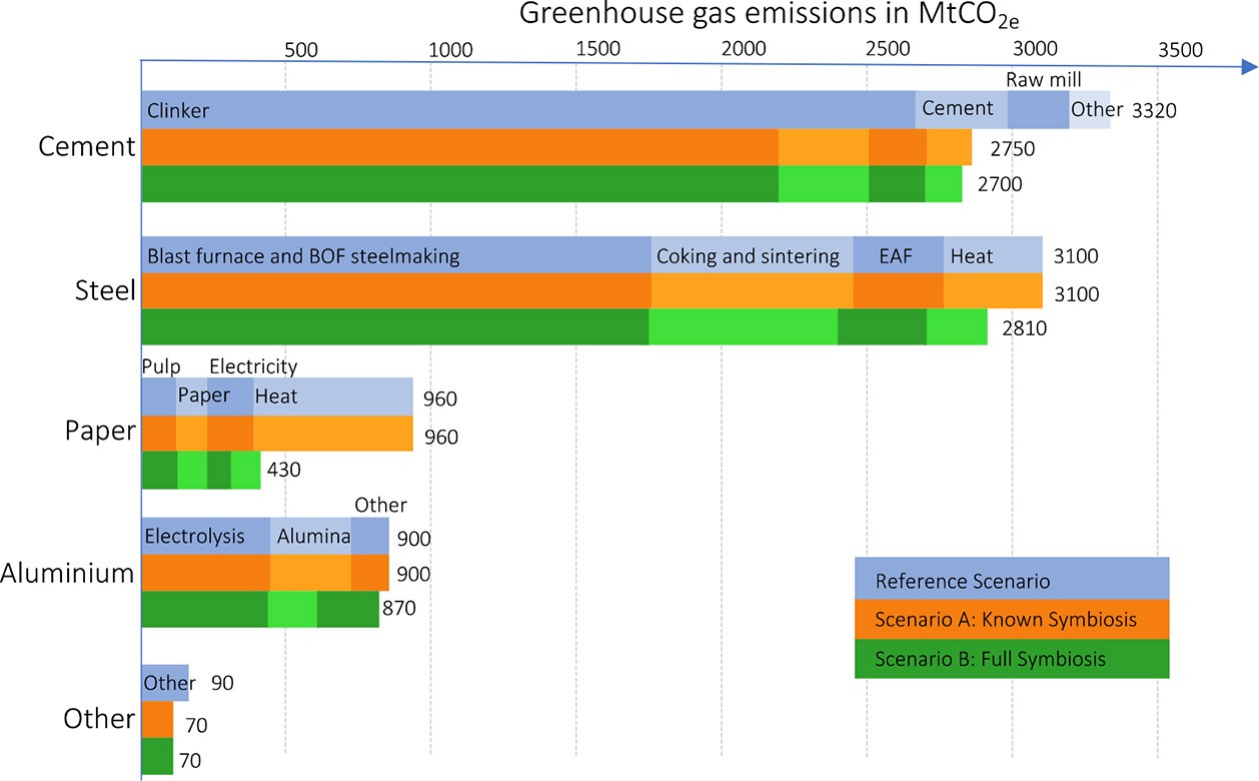
Changes in GHG emissions between the scenarios of industrial symbiosis for the bulk material production sectors.

Figure 3 shows the GHG emissions for each material across the scenarios.

In Scenario A (symbiosis with solid byproducts), the emission savings through industrial symbiosis are driven by the reduction in conventional cement production as it is substituted by other cementitious materials (slags and fly ash). The emissions from steel, aluminum, and paper production processes remain largely unchanged since no symbiosis processes using solid byproducts are known for reducing the emissions of these processes.

In Scenario B (solid byproducts, heat exchange, and ORC turbines), technologies for heat recuperation from clinker production off-gases and clinker cooling lead to a further small reduction in cement sector emissions (50 Mt CO_2e_). Steel and paper emissions are lower in Scenario B as well, driven by waste heat recovery from hot flue gases. The hot flue gases are used for preheating air for main processes as well as for electricity generation in ORC turbines.

**Figure 4:**
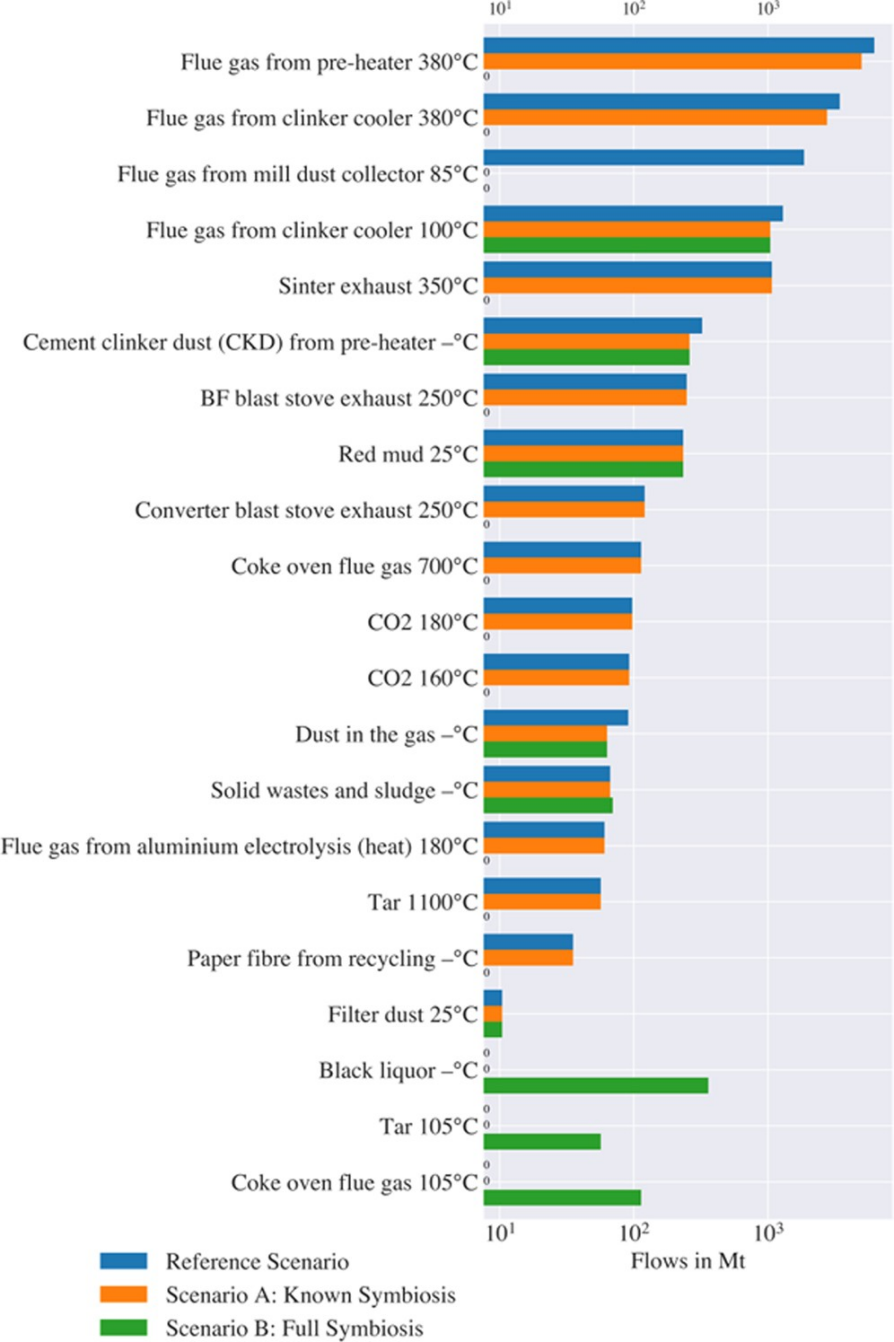
Overview of selected material flows leaving the production system as discharged flows (log scale). The substance flows (at specific temperatures) are shown for the reference scenario (blue) and symbiosis Scenarios A (orange) and B (green).

Figure 4 summarizes the flows leaving the production system as unused outputs (discharge) for the reference system and the two scenarios of symbiosis. Hot flue gas streams and slags and dust, which are discharged in the reference scenario, are used in symbiosis. Black liquor is no longer combusted in Scenario B due to the lower emission intensity of natural gas combustion. The figure draws attention to unused streams that might provide opportunities for future symbiosis including tar, dusts in flue gases, and red mud. For example, Pontikes and Angelopoulos^80^ suggest that red mud might be used as a partial replacement for cement in the future.

## 4 Discussion

The model results provide a first estimate of the global potential of industrial symbiosis in the bulk material production processes.

### 4.1 Can the Results Be Compared with Other Studies of Industrial Symbiosis?

This is the first study of the global GHG mitigation potential of industrial symbiosis. Published case studies on a country level or park level are limited to selected processes or sectors. The IPCC report indicates a potential 10–20% reduction in GHG emissions through industrial symbiosis in cement production, which could not be confirmed by this analysis. Whereas the use of secondary raw materials has a potential to decrease clinker-based emissions, the emissions from the combustion processes of (secondary) fuels still lead to emissions in the production system.

The emission reduction potential for steel production estimated by the model is lower than that in the Kawasaki symbiosis case study,^81^ in which the emission reductions for steel and cement production are estimated at around 10% of the overall park’s emissions. This is because further processes of low-temperature heat utilization for district heating are included in their case study. A significant reduction of emissions is realized through heat exchanger technologies that use hot flue gases and byproduct streams to reheat other processes and generate electricity.

Liquid and gaseous flows also provide further options for industrial symbiosis. Water cascading networks and other liquid byproducts were not included in this analysis since they contribute little to GHG emissions.^82^ Recent studies highlight significant potentials for freshwater savings^83^ but rising emissions from sludge treatment and transport.^18^ The replacement of steam generation from black liquor and blast furnace gas electricity generation with natural gas could further reduce emissions due to more efficient combustion. In that case, other uses for the byproducts have to be found. Flue gas streams containing carbon dioxide (e.g., from the blast furnaces or calcination) could be used in downstream processes and chemical reactions with the so-called carbon capture and utilization (CCU) processes. There are some case studies assessing the options for using blast furnace flue gases from steel production sites for the synthesis of chemicals.^84^ Since the CCU technologies are not yet widely deployed and no experience with their large-scale deployment exists, they were not included in Scenario B. The impact of fuel switch to natural gas and other options for utilizing the flue gases could be explored in further research.

### 4.2 How Much of the Predicted Mitigation Could Be Realized in Practice?

The global model used in this analysis relies on stylized processes, assumes no geographical boundaries to process reconfiguration and does not reflect global variations in processes. These are strong assumptions, which allow the calculation of a theoretical maximum potential for GHG emissions mitigation. The overall physical flows that can be exchanged between factories and the realizable potential of symbiosis are considerably smaller: In some locations, it might not be possible to transport byproducts between processes and the demand for some products will not match supply and there may be constraints due to the quality of the materials used as secondary byproducts. Domenech et al.^85^ analyzed the geographical constraints of industrial symbiosis in Europe and find that besides the physical distance between sites, the varying incentives and different legislative issues make the transport and utilization of waste across country boundaries difficult.

The results also depend on deployment rates for technologies of waste utilization and heat recovery. Whereas the use of the solid wastes and slags from steel production are already in practice and economic,^9^ thermoelectric waste heat recovery operates only in niche markets at present^86^ and has not been deployed at a large scale.^87^ A recent study by Nelson and Allwood^88^ analyzed 12 technological and social transitions and their time scale of technology deployment. The deployment of new energy technologies may initially grow at an exponential rate but soon stabilizes to a more linear rate due to constraints from, for example, capital availability, political will, or regulatory approval. Hence, even if ORC and additional heat exchanger technologies are introduced at scale in the industry, it might take several decades to realize the potential predicted in the results in Section 3. Additionally, the realization of the full potential can only be achieved with a redesign of existing production sites.

The goal of the present analysis was to identify a maximum potential contribution of industrial symbiosis for greenhouse gas emission mitigation, regardless of economic constraints. For assessing the economic potential, the costs for waste production, the quality differences between waste streams, and their implications for treatment and processing must be considered along with capital and transition costs. These economic constraints might strongly reduce the predicted potential for industrial symbiosis.

In some countries, it is possible that industrial symbiosis could have a greater benefit. For example, a review of potentials for reducing GHG emissions in Chinese steel production (including some symbiosis technologies) finds a potential for reducing GHG emissions by up to 40%.^89^ Similarly, there could be opportunities for reducing specific emissions per tonne of steel in regions with an overall increase in primary production like India.^90^ National potentials of symbiosis could be made on a national level using available data on specific production sites and current utilization rates of byproducts similar to this analysis or the symbiosis potential on a cluster level^91^ or national level.^17^

### 4.3 What Are the Implications for Immediate Actions for Industrial Emission Mitigation?

The results demonstrate that if industrial symbiosis is to be promoted, the focus should be given to (a) intensified utilization of other cementitious materials and (b) flue gas heat recuperation for electricity generation and heat exchange.

The potential benefit from the use of these apparently wasted cementitious materials is limited since not all clinkers can be replaced with the currently available cementitious materials. Furthermore, the production of granulated blast furnace slag and fly ash are the byproducts of emission-intensive processes, so availability will reduce over time with the closure and replacement of these processes. There are several byproducts that leave the production system as unused outputs. These include electric arc furnace dust and bauxite residues and red mud (120 Mt). Although some pilot projects already exist, they are currently not used at a large scale.^53^ Some case studies suggest that bauxite residues^80^ as well as electric arc furnace dust^92^ can be used in the construction sector in asphalt concrete mixtures.

The results suggest that a significant potential of industrial symbiosis for climate mitigation lies in organic Rankine cycle electricity generation. A review on ORC turbines by Loni et al.^93^ describing case studies of their implementation predicts a payback period of 3–6 years, depending on the time-matching of available heat and electricity demand. However, large-scale adoption is yet to occur, and given the short time available to deliver on global pledges for net-zero emissions, it seems unlikely that ORCs can make a substantial contribution.

Although the literature claims the importance of industrial symbiosis for GHG mitigation, the model of this paper does not support this claim for bulk material production processes. Under the strong assumptions of replumbing of the industrial system, some mitigation is possible. However, it arises mainly from the substitution of cement for solid byproducts from aluminum and blast furnace steel production, which will likely be phased out due to their incompatibility with net-zero production. The decarbonization of process heat and heat recovery, e.g., through a fuel switch away from coal and gas and from the deployment of ORC electricity generation provides additional opportunities for emission mitigation. However, these activities are already pursued and ORC turbines have yet to be adopted at scale. A focus on reconfiguring the current system of byproducts (with the little overall effect on GHG emissions) instead of supporting the development and deployment of other low-carbon alternatives might lead to a lock-in effect in high-carbon infrastructure and dependencies on their byproducts. The conclusion of this paper is therefore that industrial symbiosis within the bulk material process should not be considered an important contributor to climate mitigation.

## Supporting Information Available

More detailed information on the processes, production recipes used, and design of the global symbiosis model (PDF)
