# Supplementary material for: What Contribution Could Industrial Symbiosis Make to Mitigating Industrial Greenhouse Gas (GHG) Emissions in Bulk Material Production?
Source: Environ Sci Technol. 2022 Jun 30;56(14):10269–78. doi: 10.1021/acs.est.2c01753 (PMC9301909; doi:10.1021/acs.est.2c01753)
Supplement: Supplementary file 1 — es2c01753_si_001.pdf [file es2c01753_si_001.pdf]

WHAT CONTRIBUTION COULD INDUSTRIAL SYMBIOSIS MAKE TO MITIGATING INDUSTRIAL GREENHOUSE GAS (GHG) EMISSIONS IN BULK MATERIAL PRODUCTION?

LUKAS GAST[1], ANDRÉ CABRERA SERRENHO[1], JULIAN M ALLWOOD[1]

[1]*Department of Engineering, University of Cambridge, Trumpington Street, Cambridge CB2 1PZ, United Kingdom.*

Supporting Information

Table of Contents

1 Material flow charts 4
1.1 Steel production 4
1.2 Cement production 6
1.3 Aluminium production 7
1.4 Pulp and paper production 9
2 Process recipes 11
2.1 Main industrial production processes 13
2.1.1 Steel production 14
2.1.2 Cement production 16
2.1.3 Aluminium production 18
2.1.4 Paper production 20
2.2 Energy processes 22
2.2.1 Electricity generation 22
2.2.2 Steam generation 23
2.3 Options for industrial symbiosis 24
2.3.1 Utilisation of solid by-products as inputs 25
2.3.2 Waste heat recovery 26
2.3.3 Solid waste as a fuel 29
2.4 Summary of the processes in the model 30
3 Model implementation 35
4 Sensitivity analysis 36
5 References 38

Table of Figures

Figure S 1: Process chart of BF steel production in the model 4
Figure S 2: Process chart of EAF steel production in the model 5
Figure S 3: Process chart of cement production in the model 6
Figure S 4: Process chart of primary aluminium production in the model 7
Figure S 5: Process chart of secondary aluminium production in the model 8
Figure S 6: Process chart of primary paper production in the model 9
Figure S 7: Process chart of paper production in the model 10
Figure S 8: Process for average global electricity generation 22
Figure S 9: Organic Rankine Cycle (ORC) turbine used in the model 27
Figure S 10: Overview of the model in python 35
Figure S 11: Results for changes in emission factors of the processes 36
Figure S 12: Results for relative changes in emission factors of the processes 37
Figure S 13: Sectoral GHG emissions in cement production in scenario A 37

Table of Tables

Table S 1: Parameters of the production recipes 11
Table S 2: Global material demand in 2017 12
Table S 3: Constraints of scrap supply 12
Table S 4: Overview of the sources used for the production recipes 13
Table S 5: Production recipe for coking 14
Table S 6: Production recipe for sintering 14
Table S 7: Production recipe for blast furnace (BF) ironmaking 14
Table S 8: Production recipe for basic oxygen furnace (BOF) steelmaking 15
Table S 9: Production recipe for electric arc furnace (EAF) steelmaking 15
Table S 10: Production recipe for raw meal production 16
Table S 11: Production recipe for clinker production 17
Table S 12: Production recipe for average cement production 17
Table S 13: Production recipe for alumina 18
Table S 14: Production recipe for aluminium 18
Table S 15: Production recipe for aluminium ingot 19
Table S 16: Production recipe for aluminium ingot from recycling (remelting) 19
Table S 17: Production recipe for pulp making 20
Table S 18: Production recipe for paper making 20
Table S 19: Production recipe for paper from recycling 21
Table S 20: Greenhouse gas (GHG) emissions from electricity generation 22
Table S 21: Overview of processes for industrial steam generation used 23
Table S 22: Overview of the scenarios of industrial symbiosis 24
Table S 23: Flows and options for industrial symbiosis from the industrial by-products 25
Table S 24: Cement production for the scenarios with higher slag and fly ash use 25
Table S 25: Options for waste heat recovery 28
Table S 26: Full list of processes and brief description 30

1 Material flow charts

The process flow charts in the following sections represent the stylised production routes for assessing the industrial symbiosis potential. Each process is represented with a box and the main output flow is visualised with a circle. The processes which only operate in the two scenarios of industrial symbiosis are highlighted with colours: orange for processes operating in both scenarios and green for those added only in scenario B (full symbiosis).

1.1 Steel production

The process route for the blast furnace steel production used in the model is depicted in Figure S 1.

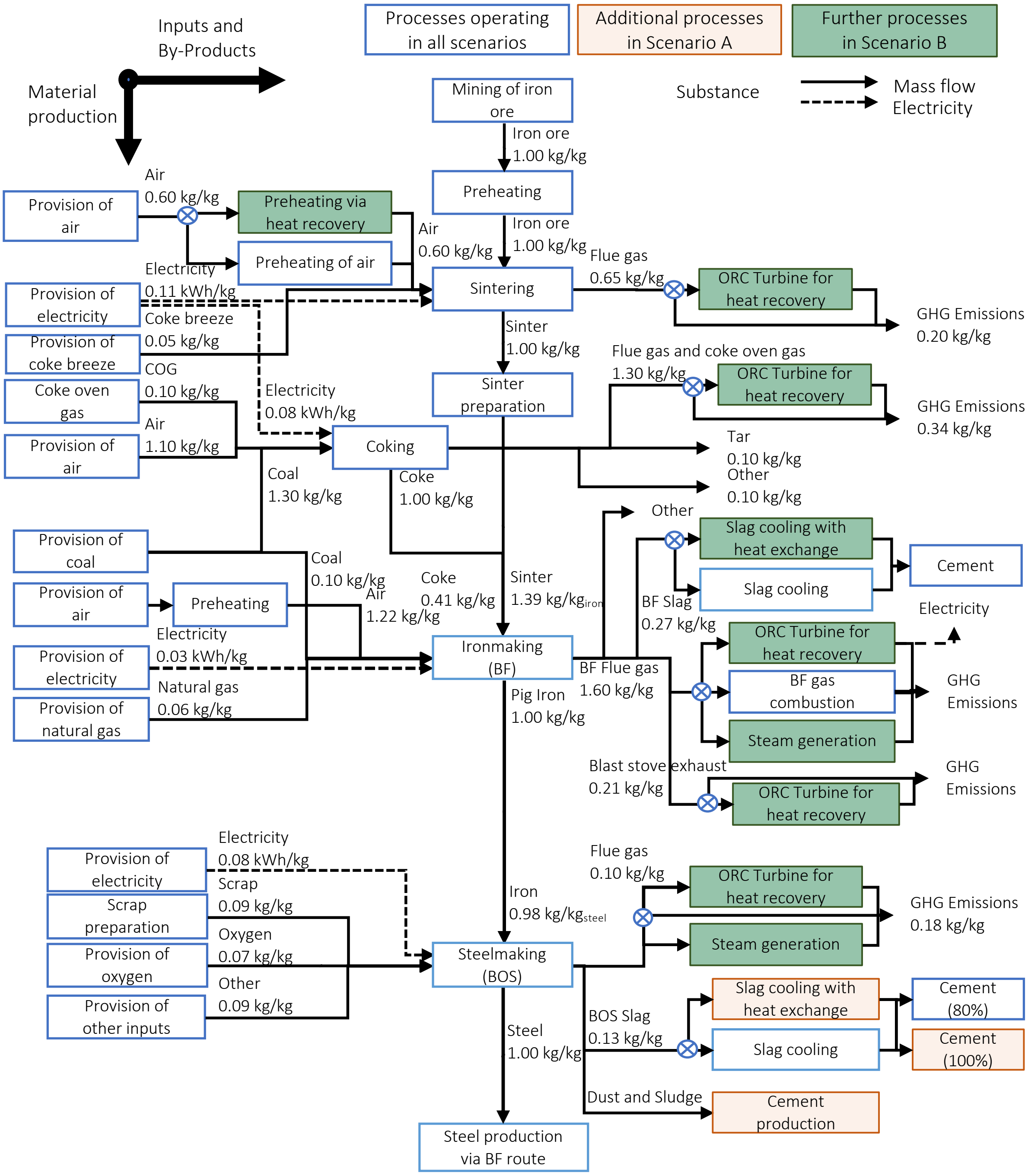

Figure S 1: **Process chart of BF steel production in the model**

The process route for the electric arc furnace steel production used in the model is depicted in Figure S 2.

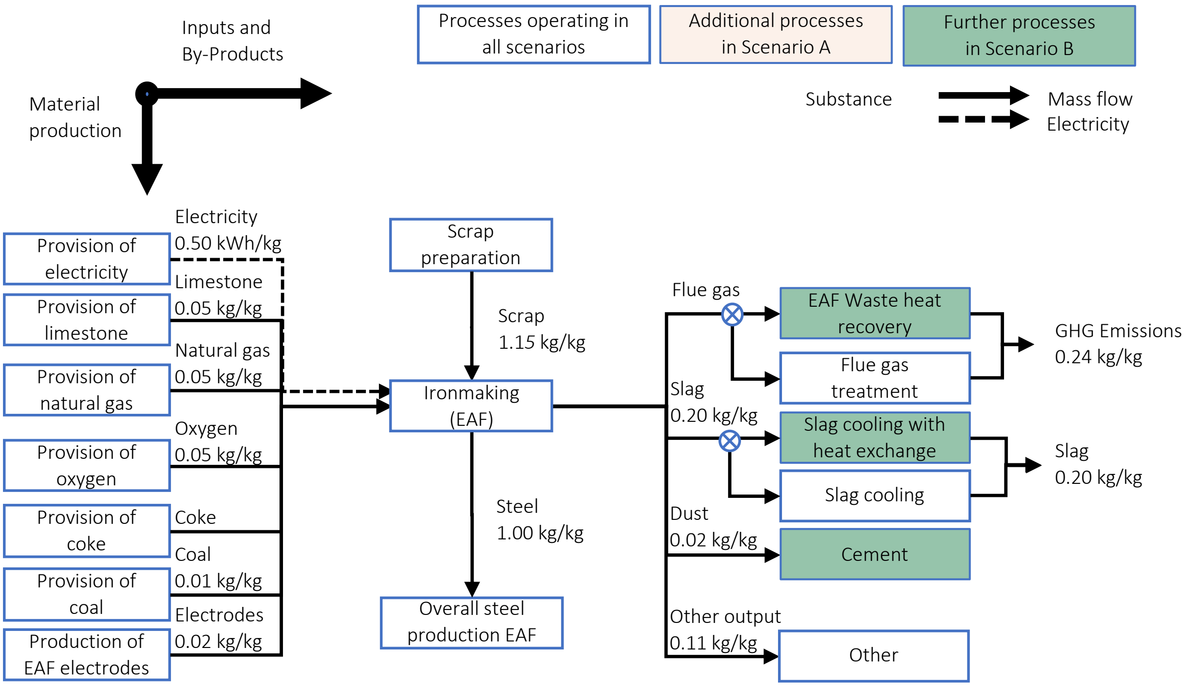

Figure S 2: Process chart of EAF steel production in the model

1.2 Cement production

The process route for the integrated cement production used in the model is depicted in Figure S 3.

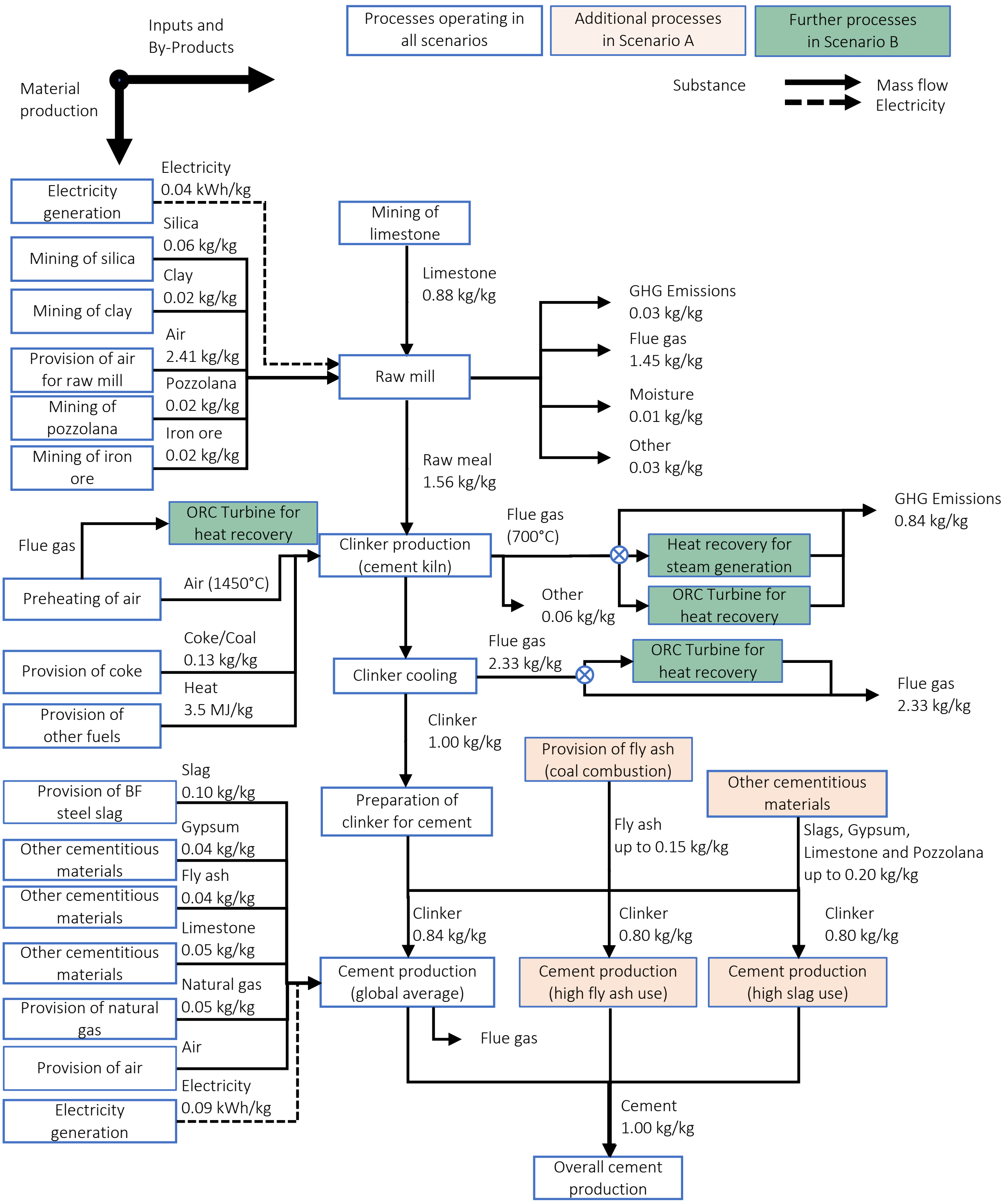

Figure S 3: Process chart of cement production in the model

1.3 Aluminium production

The process route for the aluminium production used in the model is depicted in Figure S 4.

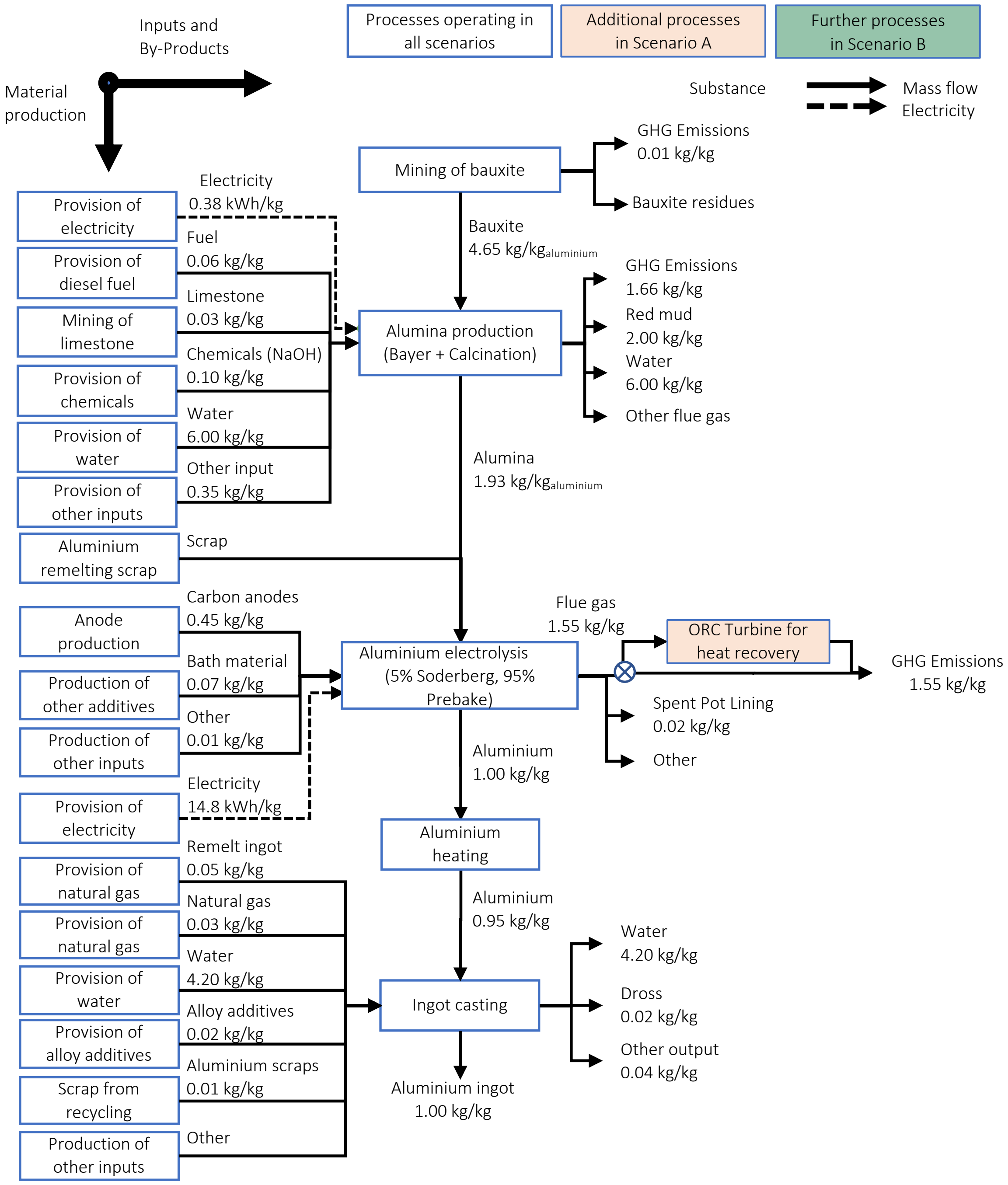

Figure S 4: Process chart of primary aluminium production in the model

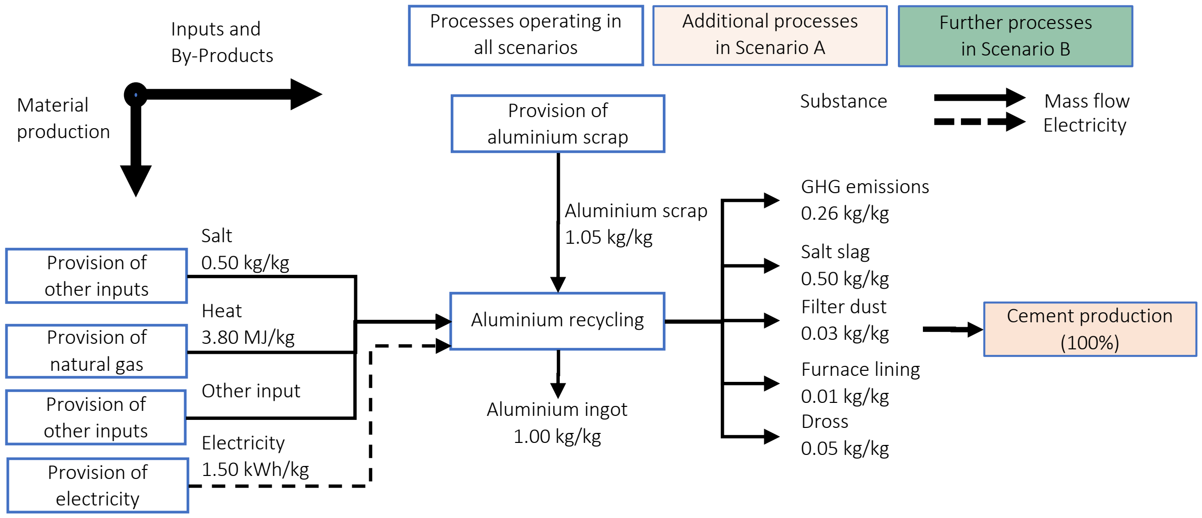

Figure S 5: Process chart of secondary aluminium production in the model

1.4 Pulp and paper production

The process route for the primary paper production used in the model are summarised in Figure S 6 and secondary route in Figure S 7.

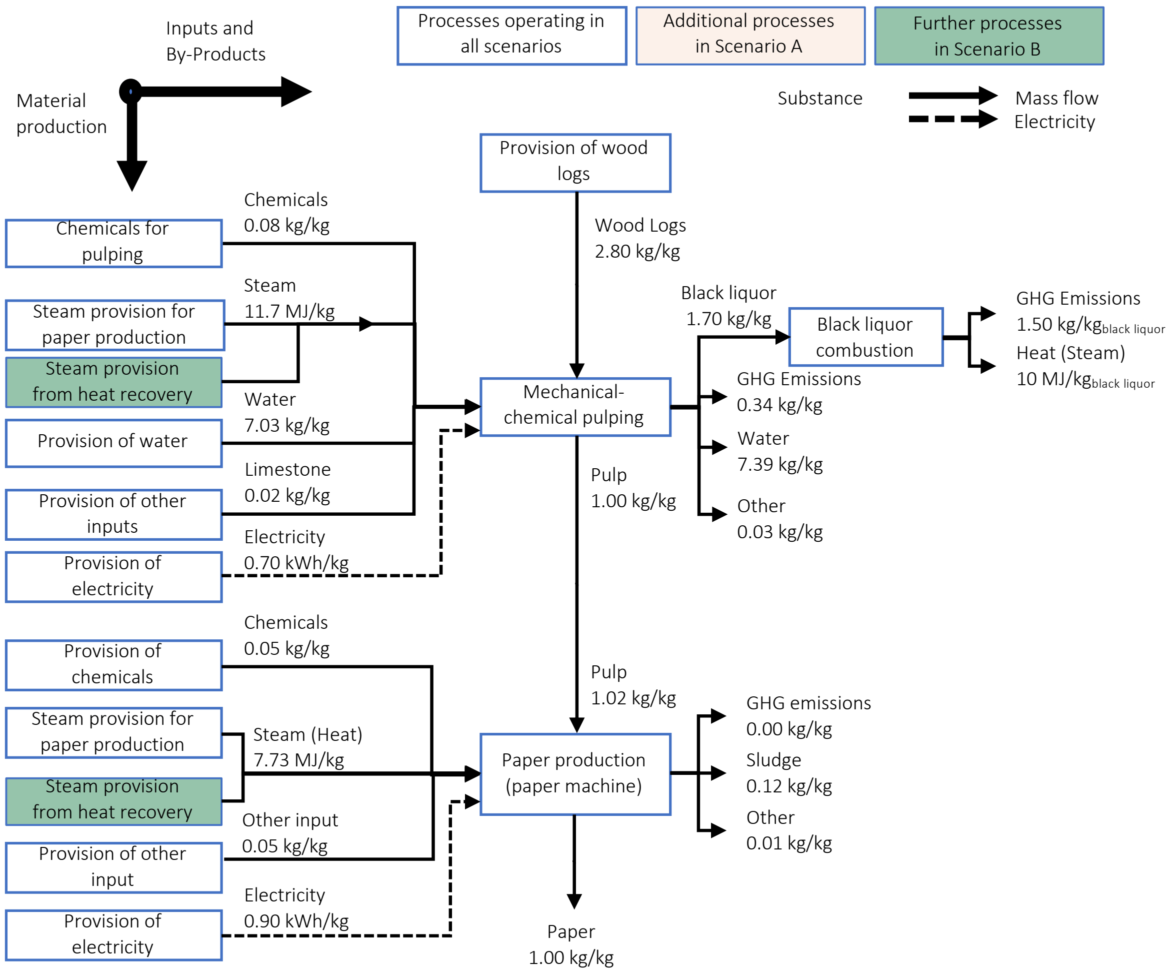

Figure S 6: Process chart of primary paper production in the model

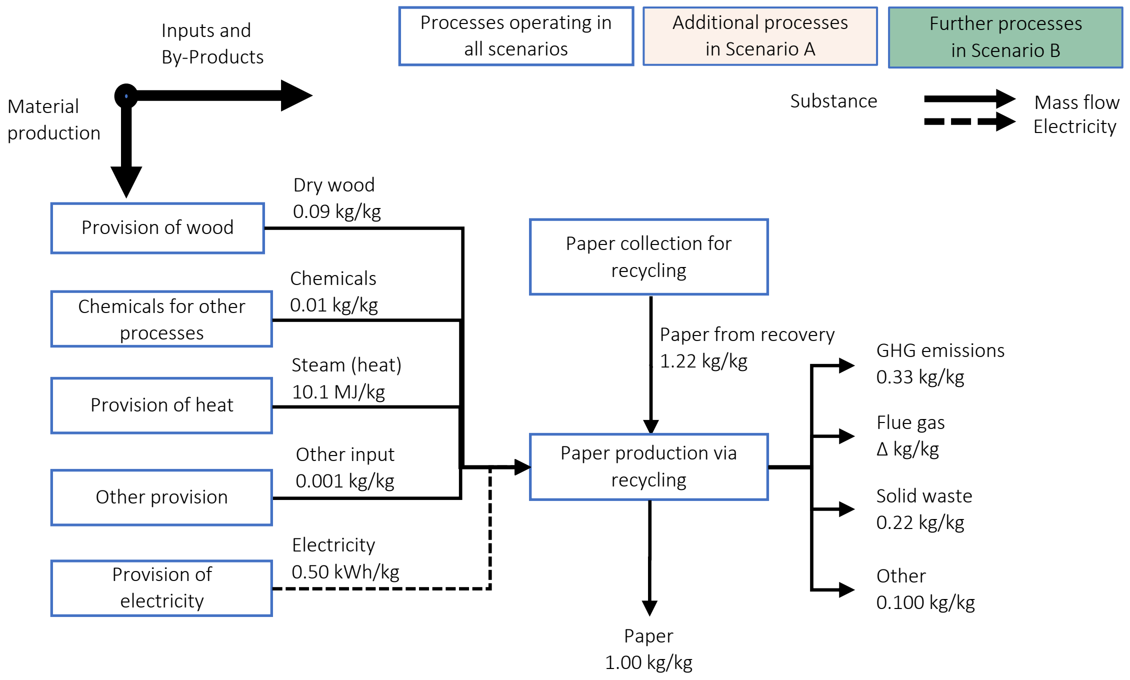

Figure S 7: Process chart of paper production in the model

2 Process recipes

The production recipes of the processes used in the model are summarised in this section in tables with material flows and further information. The production recipes were used for the production coefficient matrix for the production system.

The production processes were divided in four categories:

1. Main industrial processes: the main bulk material production processes and the processes that produce the inputs of them.
2. Energy processes for providing heat and electricity: processes for steam and electricity generation.
3. Industrial symbiosis processes for preparing materials for utilisation and combustion: processes that turn one by-product into a usable input of another process.
4. Processes for providing resources from nature and utilising by-products from industry: extraction processes (e.g. mining) and processes in other industries (e.g. chemicals), that provide the inputs needed for the major bulk material production processes.

During the data and literature review, the production recipes were collected for all major processes and sub-processes. The main industrial processes consist of several sub-processes which are summarised in the first section of this supplementary information. The sub-processes provide all inputs required for producing the final material along the process chain. The key parameters are summarised in Table S 1.

Table S 1: Parameters of the production recipes

Name	Label	Example	Description
Flow	stream_title	"Iron"	Name of the physical flow, e.g. "BF_Iron".
Temperature	temperature	300°C	Temperature of the flow in °C; hot flows can be used in heat exchangers.
Coefficient	avg_coefficient	0.1	Production coefficient for the given process, e.g. 0.1 kg/kg output.
Unit	stream_unit	kg/kg, kWh/kg	All physical flows are reported in kg/kg; kWh is used for electricity and MJ/kg for heat demand.
Direction	stream_direction	Input or output	Production coefficient is negative for inputs and positive for outputs.
Coefficient	avg_recipe_source	0.1	
Reference	source	Author (Year)	Reference(s) for the production recipes; see Table S 4 for detailed overview.
Comment	comment	Text and description	Additional information on the stream and calculations.

The global material production for primary and secondary production in 2017 is used in the model for the global material demand ($\hat{y}$). It is summarised in Table S 2.

Table S 2: Global material demand in 2017

Material Demand (Mt)	2017	Unit	Source
Blast furnace steel	1207	Mt	World Steel Association (2018a)
EAF steel	473	Mt	World Steel Association (2018a)
Cement	4050	Mt	Statista (2019)
Aluminium (primary)	63	Mt	World Aluminium (2021)
Aluminium (from scrap)	29	Mt	World Aluminium (2021)
Paper	420	Mt	Statista (2020)
Paper (primary prod.)	263	Mt	Statista (2020)

The constraints for the availability of scrap and secondary materials used in the model as finite entries in vector $\boldsymbol{c}$ are summarised in Table S 3.

Table S 3: Constraints of scrap supply

Secondary supply	2017	Unit	Source	Comment
EAF Scrap	555	Mt	World Steel Association (2018)	Input required for producing 473 Mt EAF steel as input.
Aluminium Scrap	30	Mt	World Aluminium (2021)	32% of secondary production.
Paper recycling	233	Mt	FAO (2021)	Global amount of recovered paper in 2017.
Fly ash	215	Mt	IEA (2020), Ritter (2016)	IEA (2020) reports a share of 5.3% fly ash in cement in 2019. Using this value for 2017 production: 4050 Mt * 0.053% = 214.65 Mt fly ash use for the reference scenario. For the symbiosis scenario, the constraint is removed (up to the maximum available amount of 1000 Mt).

2.1 Main industrial production processes

The following sections contain the production recipes data tables used in the global production model. Table S 4 summarises the processes and sources used for the production recipes.

Table S 4: Overview of the sources used for the production recipes

Sector	Explanation of data used	Data sources
Steel	Global average flow data for BF steel production and EAF production from a representative set of steel production sites using data from World Steel Association. Additional information from the Best Available Technologies (BAT) documents by the European Commission was used for missing flows.	[5], [6], [7], [8], [9], [10], [11], [12].
Cement	Survey data collected by the GNR initiative and additional information on flows and temperatures from peer-reviewed case studies and technical reports on the use of cementitious materials.	[13], [14], [15], [16], [17], [18], [19].
Aluminium	Data on global and European Aluminium flows from the World Aluminium Association and additional information on waste heat recovery potentials from industrial case studies.	[20], [21], [22], [23], [24], [25].
Paper	Process-level data from the BAT documents, two peer-reviewed analyses of the material flows in paper production systems and emissions data from LCI database Ecoinvent.	[26], [27], [28], [3], [29], [30], [31], [32], [12].

For steel mass flows, the global average steel resource data for BF and EAF steel production from Gonzalez Hernandez et al.[6] was used. The flow and temperature information in McBrien et al.[5] was used for the blast furnace route of steel production. For electric arc furnaces, the balanced mass flows from Krassnig et al. (2007) were used. Further information on the electricity demand and flows of sensible heat in flue gases from EAF was taken from the BAT documents[7] and information on GHG emissions from Pardo and Moya.[10]

For aluminium production processes, mass flow information was used from Balomenos et al. (2017) and temperature information was taken from Nowicki and Gosselin (2012). The information on by-product flows like bauxite residues were taken from World Aluminium (2020). The BAT document from the European Commission[22] was used for a simplified reference process for secondary aluminium production.

For cement production, the data for global average production and its inputs was taken from GNR (2017) and two further processes with a higher intake of cementitious materials (fly ash and slags) were added based on the processes described in Scrivener et al. (2016) and blended cements used in Huntzinger and Eatmon (2009). Information on clinker mass balances and temperature levels of the flows for heat recovery was taken from Gao et al. (2016).

The mass flows for paper production were taken from the BAT[28], the life-cycle inventory in Corcelli et al.[26] and for paper recycling from Christensen and Damgaard.[27] The GHG emissions factors were taken from the BAT documents and for pulp production from the data provided in the Ecoinvent database.[29]

2.1.1 Steel production

Table S 5: Production recipe for coking

stream_title	T	avg_coefficient	stream_unit	stream_direction	stream_type
Coal	1100	1.30	kg/kg	input	material
COG	1100	0.10	kg/kg	input	material
Air	1100	1.10	kg/kg	input	material
Electricity		0.08	kWh/kg	input	electricity
Coke	1100	1.00	kg/kg	output	material
COG	700	0.20	kg/kg	output	material
Tar	1100	0.10	kg/kg	output	material
Flue gas	250	1.10	kg/kg	output	material
Other_coking	1100	0.10	kg/kg	output	material
GHG_emissions		0.34	kg/kg	output	GHG

Table S 6: Production recipe for sintering

stream_title	T	avg_coefficient	stream_unit	stream_direction	stream_type
Iron ore	1300	1.00	kg/kg	input	material
Coke breeze	1300	0.05	kg/kg	input	material
Combustion Air	1300	0.60	kg/kg	input	material
Electricity		0.11	kWh/kg	input	electricity
Sinter	700	1.00	kg/kg	output	material
Flue gas	350	0.65	kg/kg	output	material
GHG_emissions		0.20	kg/kg	output	GHG

Table S 7: Production recipe for blast furnace (BF) ironmaking

stream_title	T	avg_coefficient	stream_unit	stream_direction	stream_type
Coke	1200	0.41	kg/kg	input	material
Coal	1200	0.10	kg/kg	input	material
Sinter	1200	1.39	kg/kg	input	material
Air	1180	1.22	kg/kg	input	material
Natural gas	1180	0.06	kg/kg	input	material
Electricity	0	0.03	kWh/kg	input	electricity
Chemical_energy	–	12.30	MJ/kg	input	material
Pig iron	1500	1.00	kg/kg	output	material
BF CO	180	0.32	kg/kg	output	material
BF CO2	180	0.32	kg/kg	output	material
BF Gas Other	180	0.96	kg/kg	output	material
Slag	1500	0.28	kg/kg	output	material
Blast stove exhaust	250	0.21	kg/kg	output	material
Imbalance		0.04	kg/kg	output	material
GHG_emissions		1.21	kg/kg	output	GHG

Table S 8: Production recipe for basic oxygen furnace (BOF) steelmaking

stream_title	T	avg_coefficient	stream_unit	stream_direction	stream_type
Pig iron	1700	0.98	kg/kg	input	material
Scrap	1700	0.09	kg/kg	input	material
Oxygen	1700	0.07	kg/kg	input	material
Other_steelmaking		0.09	kg/kg	input	material
Electricity		0.08	kWh/kg	input	electricity
BOF exhaust	1700	0.10	kg/kg	output	material
Slag	1700	0.13	kg/kg	output	material
Steel	1700	1.00	kg/kg	output	material
GHG_emissions		0.18	kg/kg	output	GHG

Table S 9: Production recipe for electric arc furnace (EAF) steelmaking

stream_title	T	avg_coefficient	stream_unit	stream_direction	stream_type
Limestone	25	0.05	kg/kg	input	material
Coal	25	0.01	kg/kg	input	material
Scrap	25	1.15	kg/kg	input	material
Natural gas	25	0.05	kg/kg	input	material
Oxygen	25	0.05	kg/kg	input	material
Electrode	25	0.02	kg/kg	input	material
Electricity		0.51	kWh/kg	input	electricity
Other input	25	0.10	kg/kg	input	imbalance
Dust	25	0.02	kg/kg	output	material
Steel	700	1.00	kg/kg	output	material
Slag		0.20	kg/kg	output	material
Flue gas heat	1000	0.50	MJ/kg	output	energy
CO2		0.24	kg/kg	output	material
GHG_emissions		0.24	kg/kg	output	GHG

2.1.2 Cement production

The production of cement consists of the processes for raw meal, clinker and cement production.

Table S 10: Production recipe for raw meal production

stream_title	T	avg_coefficient	stream_unit	stream_direction	stream_type
Air	25	1.01	kg/kg	input	material
Limestone	25	0.88	kg/kg	input	material
Air to roller crusher	25	0.35	kg/kg	input	material
Silica input	25	0.06	kg/kg	input	material
Leaking air	25	0.06	kg/kg	input	material
Air to dust collection	25	0.03	kg/kg	input	material
Iron ore	25	0.02	kg/kg	input	material
Clay input	25	0.02	kg/kg	input	material
Dust in the gas	25	0.02	kg/kg	input	material
Moisture	25	0.01	kg/kg	input	material
Electricity		0.04	kWh/kg	input	electricity
Flue gas	100	1.45	kg/kg	output	material
Raw meal	100	1.00	kg/kg	output	material
Other output	100	0.03	kg/kg	output	material
Moisture in raw meal	100	0.01	kg/kg	output	material
GHG_emissions		0.03	kg/kg	output	GHG

Table S 11: Production recipe for clinker production

stream_title	T	avg_ coefficient	stream_ unit	stream_ direction	stream_ type
Raw meal		1.56	kg/kg	input	material
Coal for clinker production		0.13	kg/kg	input	material
Heat requirement		3.50	MJ/kg	input	energy
Primary air into the kiln		0.05	kg/kg	input	material
Air into kiln with coal		0.02	kg/kg	input	material
Air into calciner with coal		0.04	kg/kg	input	material
Air into cooler		2.33	kg/kg	input	material
Air leaking into the kiln system		0.56	kg/kg	input	material
Clinker		1.00	kg/kg	output	material
Waste gas from pre-heater	380	1.92	kg/kg	output	material
CKD from pre-heater waste gas		0.10	kg/kg	output	material
Flue gas from clinker cooler to WHRSG	360	1.06	kg/kg	output	material
Low temperature flue gas to WHRSG from cooler		0.40	kg/kg	output	material
Flue gas from cooler to coal mill		0.14	kg/kg	output	material
CKD from cooler high temperature gas		0.01	kg/kg	output	material
Other output		0.06	kg/kg	output	material
Calcination emissions		0.55	kg/kg	output	material
Fuel emissions		0.30	kg/kg	output	material
GHG_emissions		0.84	kg/kg	output	GHG

Table S 12: Production recipe for average cement production

stream_title	T	avg_ coefficient	stream_ unit	stream_ direction	stream_ type
Limestone		0.06	kg/kg	input	material
Natural Pozzolana		0.02	kg/kg	input	material
Air		2.41	kg/kg	input	material
Natural gas		0.05	kg/kg	input	material
Slag		0.10	kg/kg	input	material
Fly ash		0.04	kg/kg	input	material
Moisture		0.01	kg/kg	input	material
Clinker		0.84	kg/kg	input	material
Gypsum		0.04	kg/kg	input	material
Electricity		0.09	kWh/kg	input	electricity
Gas from separator dust collector		1.94	kg/kg	output	material
Gas from mill dust collector		0.46	kg/kg	output	material
Cement		1.00	kg/kg	output	material
Other output		0.16	kg/kg	output	material
GHG_emissions		0.20	kg/kg	output	GHG

2.1.3 Aluminium production

For aluminium production, the data provided by World Aluminium[33] and World Aluminium[34] was used for the major mass flows. The production data was extended with the input-balances provided by European Commission[22] and case studies of aluminium production by Balomenos et al.[35]

Table S 13: Production recipe for alumina

stream_title	T	avg_coefficient	stream_unit	stream_direction	stream_type
Bauxite	25	2.40	kg/kg	input	material
NaOH	25	0.05	kg/kg	input	material
Limestone	25	0.03	kg/kg	input	material
Other input	25	0.93	kg/kg	input	imbalance
Water	25	6.00	kg/kg	input	material
Electricity		0.20	kWh/kg	input	electricity
Diesel fuel	25	0.28	kg/kg	input	material
Red mud	180	1.00	kg/kg	output	material
Water	40	6.00	kg/kg	output	material
Steam	180	0.86	kg/kg	output	material
CO2	180	0.83	kg/kg	output	material
Alumina	180	1.00	kg/kg	output	material
GHG_emissions		0.83	kg/kg	output	GHG

Table S 14: Production recipe for aluminium

stream_title	T	avg_coefficient	stream_unit	stream_direction	stream_type
Alumina		1.93	kg/kg	input	material
Carbon Anodes		0.45	kg/kg	input	material
Bath material		0.07	kg/kg	input	material
Electricity		14.80	kWh/kg	input	energy
Other input		0.06	kg/kg	input	material
Aluminium	960	1.00	kg/kg	output	material
CO2	160	1.53	kg/kg	output	material
Waste heat in flue gas	160	0.900	kWh/kg	output	material
Spent Pot Lining (SPL)	960	0.020	kg/kg	output	material
GHG_emissions		4.86	kg/kg	output	GHG

Table S 15: Production recipe for aluminium ingot

stream_title	T	avg_ coefficient	stream_ unit	stream_ direction	stream_ type
Water	25	4.25	kg/kg	output	water
Alloy additives	25	0.02	kg/kg	input	material
Aluminium scraps	25	0.02	kg/kg	input	material
Remelt ingot	25	0.06	kg/kg	input	material
Electricity		0.07	kWh/kg	input	electricity
Liquid aluminium (from electrolysis)	960	0.94	kg/kg	input	material
Natural gas	25	0.03	kg/kg	input	energy
Water	300	4.20	kg/kg	output	water
Dross	300	0.02	kg/kg	output	material
Other output		0.10	kg/kg	output	material
Aluminium ingot	300	1.00	kg/kg	output	material
GHG_emissions		0.13	kg/kg	output	GHG

Table S 16: Production recipe for aluminium ingot from recycling (remelting)

stream_title	T	avg_ coefficient	stream_ unit	stream_ direction	stream_ type
Aluminium scrap	25	1.00	kg/kg	input	material
Salt	25	0.50	kg/kg	input	material
Other input	25	0.07	kg/kg	input	material
Electricity		1.50	kWh/kg	input	electricity
Heat		3.80	MJ/kg	input	energy
Salt slag	25	0.50	kg/kg	output	material
Filter dust	25	0.03	kg/kg	output	material
Other output	25	0.01	kg/kg	output	material
Dross	25	0.08	kg/kg	output	material
Aluminium ingot	25	1.00	kg/kg	output	material
GHG_emissions		0.26	kg/kg	output	GHG

2.1.4 Paper production

For paper production, a typical tissue mill in the European Union[28] was used as a reference for the energy and mass flows.

Table S 17: Production recipe for pulp making

stream_title	T	avg_ coefficient	stream_ unit	stream_ direction	stream_ type
Electricity		0.70	kWh/kg	input	electricity
Natural gas (heat)		0.03	kg/kg	input	material
Steam (heat)	400	11.7	MJ/kg	input	energy
Oxygen		0.02	kg/kg	input	material
NaOH	25	0.05	kg/kg	input	material
Water (inflow)	25	7.03	kg/kg	input	material
Wood logs	25	2.80	kg/kg	input	material
Biomass residues	25	0.43	kg/kg	input	material
Chemicals for pulping	25	0.08	kg/kg	input	material
Other input	25	0.04	kg/kg	input	material
Pulp (bleached)	25	1.00	kg/kg	output	material
Solids	25	0.01	kg/kg	output	material
Water	25	7.39	kg/kg	output	material
Black liquor	25	1.70	kg/kg	output	material
CO2	25	0.34	kg/kg	output	material
GHG_emissions		0.34	kg/kg	output	GHG

Table S 18: Production recipe for paper making

stream_title	T	avg_ coefficient	stream_ unit	stream_ direction	stream_ type
Electricity		0.90	kWh/kg	input	electricity
Chemicals		0.05	kg/kg	input	material
Other input		0.05	kg/kg	input	material
Heat_Steam		7.73	MJ/kg	input	energy
Heat_Water		0.24	MJ/kg	input	energy
Heat_Water		0.12	MJ/kg	input	energy
Pulp		1.02	kg/kg	input	material
Paper		1.00	kg/kg	output	material
Sludge		0.12	kg/kg	output	material
Other		0.02	kg/kg	output	material
GHG_emissions		0.00	kg/kg	output	GHG

Table S 19: Production recipe for paper from recycling

stream_title	temperature	avg_coefficient	stream_unit	stream_direction	stream_type
Heat for paper recycling	150	10.1	MJ/kg	input	heat
Electricity		0.50	kWh/kg	input	electricity
Dry wood	25	0.09	kg/kg	input	material
Chemicals for paper production	25	0.01	kg/kg	input	material
Paper fibre from recycling	25	1.22	kg/kg	input	material
Paper (recycling route)	25	1.00	kg/kg	output	material
Solid waste (from paper recycling)	25	0.22	kg/kg	output	material
Other output	25	0.10	kg/kg	output	material
GHG_emissions		0.33	kg/kg	output	GHG

2.2 Energy processes

Various processes for electricity generation, heat generation and heat exchange are used in the model. The following sections summarise the key processes and provide references for the values used for the stylised routes.

2.2.1 Electricity generation

There are four major processes for generating electricity. For the electricity generation in the reference scenario, the global average emissions of electricity production (0.475 kg/kWh) is used [36].

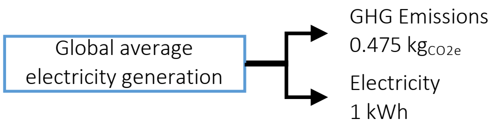

Figure S 8: Process for average global electricity generation

The GHG emissions for the processes were taken from [37] for coal, gas and biomass combustion and summarised in Table S 20.

Table S 20: Greenhouse gas (GHG) emissions from electricity generation

Process name	GHG emissions (kgCO2/kWh)	Description	Source
Global average electricity production	0.475	Average global carbon footprint from electricity generation	IEA (2019)
Electricity generation from gas	0.204	Average UK carbon footprint from gas-based electricity generation	BEIS (2019)

Blast furnace flue gas is combusted using the average heating value (2.7-4.0 MJ/Nm^3 with 20-28% carbon monoxide and 1-5% hydrogen [38]). It is assumed that all BF flue gas is combusted. The carbon footprint of 643 kg/t[39] was rounded up to 700 kg/t ([40]). Overall, the combustion is assumed to generate 0.2 kWh/Nm^3 using conventional combustion efficiency of 30% [8] and converting the MJ to kWh.

2.2.2 Steam generation

Industrial boilers are used for generating steam. Typical efficiencies are taken from ETSAP (2010) and Office of Industrial Technology (2000). Table S 21 provides a summary of the processes and their efficiency.

Table S 21: Overview of processes for industrial steam generation used

Process name	Combustion efficiency (%)	Description	Source
Gas boiler	84%	The average efficiency of 84% was chosen for steam generation from gas.	ETSAP (2010), ITEE (2000)
Coal boiler	89%	The average efficiency of 89% was chosen for steam generation from coal.	ETSAP (2010), ITEE (2000)
Black liquor combustion	80%	The average efficiency of 80% was chosen for steam generation from biomass. It is assumed that steam generation from black liquor (BL) has a similar efficiency.	Paoli and Cullen (2020)[41]

The steam requirement was usually reported in MJ/kg. For the processes, for which a direct steam flow was required, the steam flows were calculated. The data for the steam heat values was used from steam tables in Rogers and Mayhew.[42]

In the reference scenario without industrial symbiosis, current combustion processes are used for preheating substance flows. In the scenario of industrial symbiosis, further options for heat exchange between processes are added. The preheaters either directly heat up cold substance flows (e.g. through electricity or fuel) or indirectly heat processes through heat transfer from another hot fluid that is cooled down in the heat exchanger. For the heat exchangers in the model, a heat transfer efficiency 76% was used for the heat exchanger networks.[43]

2.3 Options for industrial symbiosis

How can industrial by-products be used as an input to another process? The processes that prepare and use those by-products are referred to as “processes of industrial symbiosis” in this model. There are several options for industrial symbiosis, which have been applied at different scales across different industrial processes. A literature review of major case studies of industrial symbiosis was used to obtain a database of by-product exchanges and to classify the symbiotic options for the major bulk material production processes.

Table S 22: Overview of the scenarios of industrial symbiosis

	**Reference scenario**	**Scenario A**	**Scenario B**
Material production	Global material production in 2017.		
Main processes	Production processes for major bulk materials.		
Auxiliary processes	Same auxiliary processes for providing the external inputs.		
Minimum production	Full combustion of black liquor (BL) and blast furnace (BF) gas within the production system.		
**Symbiosis processes**			
Use of secondary raw materials and by-products	Based on current (2017) global average intake, e.g. in cement production. Only a few by-product utilisation processes (representing current use).	No constraints on the maximum intake of cementitious materials (given supply constraints). More by-product utilisation processes are operating.	No constraints on the maximum intake of secondary materials. All by-product utilisation processes are operating.
Heat exchange and utilisation	Processes for heat recovery are not operating.	Processes for heat recovery are not operating.	All symbiosis processes and options for heat exchange and recovery (ORC turbines) are operating.

The symbiosis processes include processes for heat recuperation as well as material utilisation for substituting another input. In order to classify the processes, four different categories were used. These categories reflect the degree to which the technologies for by-product exchanges have been developed or applied. The categories are the following:

1) Commercially applied, processes with high technology readiness level (TRL).
2) High TRL, applied in several but not all countries.
3) Pilot projects and case studies of industrial symbiosis.
4) Not currently deployed (e.g. economic constraints) or no information on application available, literature suggests a potential for symbiosis.

The processes in category 1 were used in the reference scenario. The processes in category 2 are included in the symbiosis scenario A and the options in categories 3 and 4 in scenario B.

2.3.1 Utilisation of solid by-products as inputs

Table S 23: Flows and options for industrial symbiosis from the industrial by-products

Process Name	Flow	Description and Example	Example case study
Coal-fired power plant	Fly ash	Using fly ash as a clinker substitute in cement production.	Chertow (2012)
Mechanical-chemical pulping	Black liquor	Combustion for electricity and heat (steam) generation	Bajpai (2014)
Ironmaking (Blast Furnace)	BF slag	Using BF slag as a clinker substitute in cement production.	Chertow (2012)
Steelmaking in BOS	BOS slag	Using BOS slag as a clinker substitute in cement production.	Chertow (2012)
Steelmaking in EAF	EAF slag	Using EAF slag as a clinker substitute in cement production.	Chertow (2012)

For the reference scenario, a BF slag intake of 70% is used as a clinker substitute and no EAF slag and dusts, which represents global slag utilisation in 2010[44]. For the scenario of full symbiosis (B), a replacement of clinker with BOS and EAF slag is introduced. The maximum technical use of fly ash in Portland cement (35%) was taken from Hoenig and Twigg (2009). The cement production processes operating in the symbiosis scenarios are summarised in Table S 24.

Table S 24: Cement production for the scenarios with higher slag and fly ash use

Substance		Cement production with BF slag	Cement production with increased use of BF slag and gypsum	Cement production with fly ash
GHG_emissions	kg/kg	0.05	0.05	0.05
Electricity	kWh/kg	-0.085	-0.085	-0.085
Natural gas	kg/kg	-0.050	-0.050	-0.050
BF/BOS Slag	kg/kg	-0.25	-0.20	
Limestone	kg/kg	-0.065	-0.065	-0.065
EAF Dust	kg/kg		-0.10	
Moisture	kg/kg	-0.01	-0.01	
Clinker	kg/kg	-0.6	-0.6	-0.70
Fly ash	kg/kg	-0.035		-0.30
Gypsum	kg/kg	-0.05	-0.1	-0.05
Cement	kg/kg	1	1	1
Pozzolana and other cementitious materials	kg/kg		-0.05	

2.3.2 Waste heat recovery

In the analysis of a global symbiosis, the urban sector is included via heat recovery e.g. in Organic Rankine Cycle turbines in scenario B. The detailed inclusion of the urban sector, i.e. district heating networks was not included due to several reasons: there are practical limitations of implementing these technologies for remote production sites and the overall utilisation potential is dependent on available infrastructure, such as district heating systems which would need to be included in the emissions accounting. Some practical limitations are the following:

- **Timing**: The waste heat availability ("generation" does not necessarily match with the heat demand by households. This is especially important for batch processes, e.g. electric arc furnaces for which steam accumulators are required.[45] For periods without heat demand in summer, additional ORC turbines are needed[46] to capture the waste heat.
- **Distance**: The efficiency of heat transport and provision limits the potential demand to areas around industrial clusters: The recommended maximum distance of the pipe infrastructure between heat source and user is between 10 kilometres[47] and 15 kilometres[48] in order to avoid major heat transport losses. Hence, there are three categories of residential buildings which can use the waste heat: (1) new buildings, (2) buildings with decentralised heat generation that can be changed to district heating and (3) buildings already connected to the DH grid. Older buildings, especially Victorian houses, will most likely not profit from the DH since they are not connected to the DH network. Overall, the distance of the generation to the district heating network reduces the overall potential though some first studies estimate that the distance can be increased to a few dozen kilometres.[49] The utilisation of waste heat, however, could assessed in a more detailed geographical analysis of potentials of symbiosis.
- **Large infrastructure investments**: For district heating networks, large infrastructure investments are needed. Current approaches for life-cycle assessment of DH network, does not include emissions of the infrastructure[50]. The embodied emissions from the infrastructure are low compared to the potential savings from a more efficient generation [51].
- **Alternatives for heating:** On the heat supply side, other low-carbon technologies compete with heat provided by heat networks and affect its implementation. These are decentralised gas- or biomass-based boilers and electric heat pumps, which could have lower specific emissions than centralised gas- or oil-fired boilers.[52]
- **Regulation**: The difficulty of district heating deployment lies in the infrastructural arrangement and contractual design. Bürger et al. (2019) describe the heterogenous institutional set-up of district heating markets and discuss the challenges of transforming district heating grids.
- **Back-up capacities and functional storage**: The availability of back-up capacities for heat generation during production stops (planned and unplanned) as well as provision of heat in intermitting processes (e.g. batch-processes like the EAF) pose further challenges to the operation of DH networks. If planned carefully, the DH networks could be used as a thermal heat storage to balance intermittent renewable electricity using heat accumulators [54]. Current design of DH networks, however, allows for only limited flexibility. [55] Hence, gas- or coal-fired power plants would be needed as a back-up capacity to the heat networks. These might then have higher carbon footprints per unit of heat delivered (compared to decentralised heating), reducing the overall efficiency gains through DH networks.

For the recovery of waste heat from hot flue gas streams, Organic Ranking Cycle (ORC) turbine processes were added. These generate electricity from hot flue gases through a primary and secondary steam cycle using various fluids. The typical efficiency the ORC turbine of 15% is used.[56] Figure S 9 illustrates a representative and a simplified process design of conversion of hot flue gases to cold flue gases and electricity. The ORC turbines in the model follow the simplified process design.

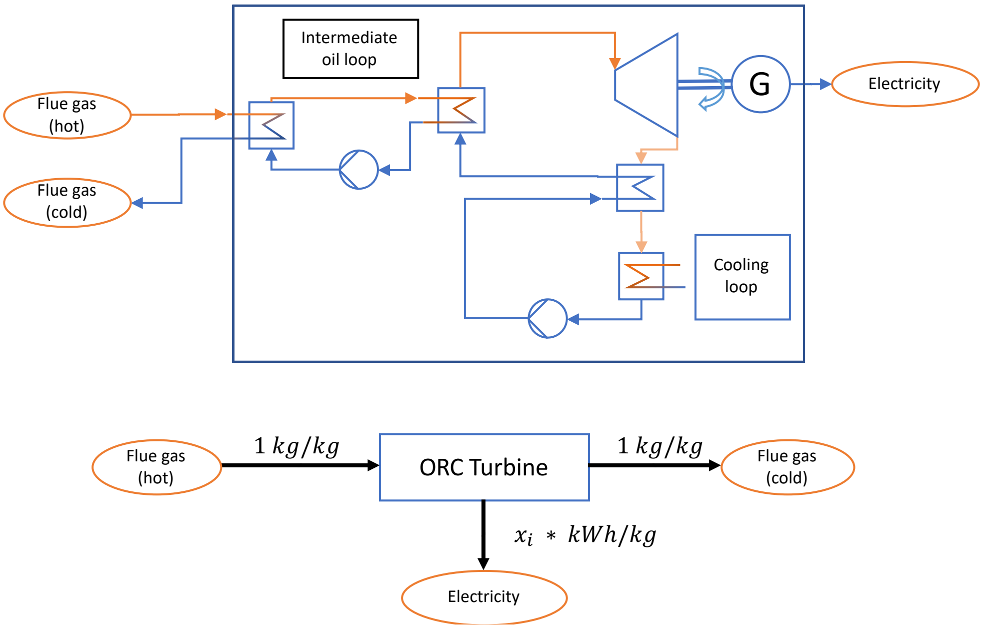

Figure S 9: Organic Rankine Cycle (ORC) turbine used in the model

For some heat recovery processes, data on available processes for heat recovery was available. These reported implementation of heat recovery options in the industrial processes were used for several flows. The processes are summarised in Table S 25 and added to the production system as "symbiosis processes" and operate in scenario B.

Table S 25: Options for waste heat recovery

Group	Process Name	Flow	Description and Example	Example case study
Aluminium	Aluminium production waste heat recovery	Flue gas	700°C flue gas converted into air (80°C) and electricity (0.9 kWh/kg).	Castelli et al. (2019)
Aluminium	Alumina production waste heat recovery	Flue gas	180°C flue gas stream used for heat recovery in ORC	Castelli et al. (2019)
Cement	Cement production	Clinker production flue gas	The ORC turbine added to the clinker production therefore generates 0.045 $kWh/kg_{clinker}$ flue gases.	Zhu (2011)
Cement	Clinker production	Flue gas	Heat recovery of hot flue gas from clinker cooler	Cao et al. (2018)
Cement	Cement production	Hot flue gases from clinker preheating	Steam generation from clinker preheater flue gas	Tsiliyannis (2018)
Steel	Sinter waste heat recovery	Sinter flue gas	Electricity generation; 0.02 kWh/kg	Li et al. (2010)
Steel	Converter flue gas waste heat recovery	Converter flue gas	Electricity generation; 0.012 kWh/kg	Li et al. (2010)
Steel	Ironmaking (Blast stove exhaust)	Flue gas	Heat recovery of hot flue gas	McBrien et al. (2016)
Steel	Converter flue gas waste heat recovery	Converter flue gas	Waste heat boiler to generate steam from BOF exhaust	McBrien et al. (2016)
Steel	Sinter waste heat recovery	Sinter flue gas	Heat recovery of hot flue gas	McBrien et al. (2016)
Steel	Blast furnace gas (180°C)	Flue gas	Heat recovery of hot flue gas with ORC	McBrien et al. (2016)
Steel	Ironmaking (Blast stove exhaust) ORC	Flue gas	Heat recovery of hot flue gas with ORC	McBrien et al. (2016)
Steel	Coke oven flue gas recovery	Coke oven flue gas	Heat recovery	McBrien et al. (2016)
Steel	Coke oven flue gas recovery	Coke oven flue gas	Heat recovery	McBrien et al. (2016)
Steel	EAF waste heat recovery	EAF waste heat	A waste-heat-recovery boiler can generate electricity offering a 7.5% reduction in EAF energy requirements.	Chan et al. (2019)
Steel	Coke oven tar recovery	Coke oven flue gas	Heat recovery	McBrien et al. (2016)

2.3.3 Solid waste as a fuel

The use of solid waste was included in the model as a fuel for cement production using current global average co-incineration of alternative fuels. An example case study using municipal solid waste as a fuel is provided by Hashimoto et al.[57] In that, they use a LCA-based substitution analysis to assess the effect of a fuel switch from coal to municipal solid waste, which have a slightly lower carbon footprint, in four scenarios of symbiosis. The carbon footprint of the fuel use is reduced from 2.58 tCO2/t (coal) to 2.44 tCO2/t (MSW), leading to an overall emissions reduction of around 44ktCO2 per year (10% of the combustion-related emissions).

If biomass fuels or alternative fuels are combusted instead of fossil fuels, the overall emissions might remain constant (or increase) while the fossil-based emissions are reduced. Figure 18 in the report by Hinkel et al.[58] visualises emissions factors and typical biomass content of different alternative fuels and contrasts them with fossil fuels currently used in cement production. The biomass content of typical fuels such as animal meal and sewage fuel is up to 100% with an emission factor of up to 110 kgCO2/GJ) The emissions factor for fossil fuels is significantly lower (55-90 kgCO2/GJ), though these emissions are solely fossil-based. If the emissions accounting from the WBCSD[59] is used, the biomass-based emissions are not allocated to cement production. Additional through MSW combustion were not attributed to the co-incineration since the model does not differentiate between biogenic and fossil-based CO2 emissions.

The analysis in this paper, however, includes all carbon dioxide emissions regardless of their origin (biomass/fossil-based). Hence, the emissions from biomass-based waste are included as normal GHG emission and additional GHG emissions reduction credits were not attributed to the processes. The theoretical potential of GHG emissions reduction reported in Hashimoto et al.[57] considers the biomass-based emissions as emissions savings since they use a LCA-based emissions factor. Hence, they estimate a larger potential for savings due to the fuel switch to MSW.

2.4 Summary of the processes in the model

The processes used in the scenarios are summarised in this section. Table S 26 summarises the processes of the production system. For each process, a short description is provided.

Table S 26: Full list of processes and brief description

Process name	Process name (model)	Description
Coking	st_coking	Produces coke required for BF steel production
Sintering	st_sintering	Produces sinter required for BF steel production
Ironmaking (Blast Furnace)	st_bf_ironmaking	Produces liquid iron for steelmaking
Ironmaking (Blast Furnace)	st_bf_ironmaking_t2	Produces liquid iron for steelmaking (with top-gas circulation)
Steelmaking (BOS)	st_bos_steelmaking	BOS Steelmaking for steel
Overall steel production via BF route	st_bf_steel_total	Overall BF steel (from different production routes)
Steelmaking (EAF)	st_eaf_steelmaking	EAF ironmaking for EAF steel
Steelmaking (EAF) with heat recovery	st_eaf_steelmaking_s20	EAF ironmaking for EAF steel with more efficient process
Overall steel production via EAF route	st_eaf_steel_total	Overall EAF steel (from different production routes)
Electricity generation (BF steel)	st_el_steel_bf	Provides electricity for BF/BOS steel production
Electricity generation (EAF steel)	st_el_steel_eaf	Provides electricity for EAF
Sinter heating	st_sinter_heating	Heating sinter for use in BF
Iron heating	st_iron_heating_fuels	Heats iron for steelmaking process
Scrap cooling	st_bf_cooling_scrap	Cooling scrap from BF route
Raw meal production in raw mill	ce_raw_mill	Produces raw meal for clinker production
Clinker production	ce_clinker_production	Produces clinker for cement production
Clinker production (T2)	ce_clinker_production_t2	Produces clinker for cement production
Cement Production (Global Average)	ce_cement_production_global_avg	Global average cement production based on GNR data
Cement Production (T1)	ce_cement_production_s30	Cement production process with by-product use 1
Cement Production (T2)	ce_cement_production_s31	Cement production process with by-product use 2
Cement Production (T3)	ce_cement_production_s32	Cement production process with by-product use 3
Preparation of clinker for cement (heating)	ce_clinker_preparation_for_cement	Overall cement production
Overall cement production	ce_total_cement_production	Preparesclinker for use in cement production
Electricity generation (cement)	ce_el_cement	Provides electricity for cement production
Alumina production	al_alumina_production	Produces alumina from bauxite
Aluminium electrolysis	al_aluminium_electrolysis	Produces liquid aluminium from alumina
Ingot casting	al_aluminium_ingot	Casting aluminium ingots from aluminim
Aluminium heating	al_aluminium_heating	Heating aluminium for casting
Aluminium cooling	al_aluminium_cooling	Process for cooling hot aluminium

Process name	Process name (model)	Description
Aluminium remelting ingot scrap	al_aluminium_remelt_ingot_scrap	Provides Aluminium scrap for electrolysis
Aluminum recycling	al_aluminium_recycling	Process for recycling aluminium (remelting)
Electricity generation (aluminium)	al_el_aluminium	Provides electricity for aluminium production
Mechanical pulping	pp_pulp_mech_chem	Mechanical and chemical pulping process for paper production
Chemical pulping	pp_paper_recycling	Paper recycling processes
Heat provision for paper production	pp_heat_paper_recycling	Provides heat/steam for paper production
Papermaking production	pp_paper_primary_route_making	Heat provision for paper recycling
Global paper demand	pp_paper_demand	Global paper demand
Primary paper production	pp_paper_primary	Overall primary paper production via primary route
Paper recovery	pp_paper_secondary	Uses pulp for producing paper
Electricity generation (paper)	pp_el_paper	Electricity generation for paper production
Electricity generation	pp_el_paper_co_generation	Electricity generation from biomass combustion
Combustion of black liquor	pp_paper_black_liquor_combustion_s51	Combustion of black liquor
Electricity generation from global average	en_el_global_avg	Average carbon footprint of global electricity generation
Electricity generation from coal	en_el_coal	Generates electricity from coal
Electricity generation from gas	en_el_gas	Generates electricity from gas
Electricity generation from BF gas combustion	en_el_bf_combustion	Generates electricity from combusting BF gas
Preheating of air	st_heat_preheating	Preheating process for air
Heat provision from natural gas	st_heat_gen_gas	Hot flue gas / steam from natural gas combustion
Steam generation from coal	pp_heat_gen_coal	Hot flue gas / steam from coal combustion
Steam generation from gas	pp_heat_gen_gas	Hot flue gas / steam from natural gas combustion
Steam generation from biomass	pp_heat_gen_biomass	Hot flue gas / steam from biomass combustion
Steam generation	pp_steam_paper1	Provides steam/heat for paper production
Steam generation	pp_steam_paper2	Provides steam/heat for paper production
Steam generation	pp_steam_paper3	Paper production steam provisio with hot flue gas streams from other processes
Heat provision for cement	cem_heat	Heat provision for cement production
Heat provision from coal (CEM)	cem_heat_gen_coal	Provides steam/heat from coal
Heat provision from natural gas (CEM)	cem_heat_gen_gas	Provides steam/heat from gas
Heat provision from coal	al_heat_gen_coal	Provides steam/heat from coal
Heat provision from natural gas	al_heat_gen_gas	Provides steam/heat from gas

Process name	Process name (model)	Description
Heat provision from coal	en_heat_gen_coal	Provides steam/heat from coal
Heat provision from natural gas	en_heat_gen_gas	Provides steam/heat from gas
Heat generation from biomass	en_heat_gen_biomass	Provides steam/heat from biomass
Heat generation from coal	en_coal_combustion_flue_gas	Hot flue gas / steam from coal combustion
Heat generation from gas	en_gas_combustion_flue_gas	Hot flue gas / steam from natural gas combustion
Heat generation from biomass	en_biomass_combustion_flue_gas	Hot flue gas / steam from biomass combustion
Flue gas heat recovery (to 40°C)	en_air_cooling	Cooling hot flue gases before emitting to atmosphere
Coal preparation for combustion	en_coal_preparation_combustion	Preparation of coal for combustion
Heat from gas	en_gas_other	Heat provision from gas
Gas for ironmaking	st_gas_ironmaking	Provision of natural gas for ironmaking
Preparation of iron ore	st_preparation_iron_ore	Preparation of iron ore for sintering
Provision of coke breeze from coke	st_coke_to_coke_breeze	Provides coke for coke breeze in steel production
Production of EAF electrodes	st_eaf_electrodes	Provides EAF electrodes
Scrap heating	st_eaf_steel_scrap_heating	Heating of scrap
Coke oven gas combustion	st_coke_oven_gas	Combustion of coke oven gas (COG)
Air provision	st_provision_coke_oven	Provison of combustion air for coke oven
Preheating process ironmaking	st_air_preheating_ironmaking	Preheating process for air in ironmaking
Oxygen provision steelmaking	st_oxygen_provision_steelmaking	Oxygen provison for steelmaking
Oxygen provision ironmaking	st_oxygen_provision_ironmaking	Oxygen provison for ironmaking
Steel production imbalances	st_imbalance	Imbalance from steel production
Air provision	ce_air_cement	Provision of air for cement production
Diesel fuel for clinker production	ce_fuel_clinker	Provision of coke for clinker production from coal. The emission factor used is 0.14 kgCO2/kgCoke (from coal)
Air provision	ce_gas_cement	Air provision for cement
Air provision	ce_air_raw_mill	Air provision for raw mill
Cement production moisture and dust	ce_raw_mill_moisture_dust	Moisture and dust from cement production
Air provision	ce_air_provision_roller_crusher	Air provision for roller crusher
Air provision	ce_air_provision_leaking_air	Air provision
Air provision	ce_air_provision_dust_collector	Air provision
Raw mill moisture and dust	ce_air_provision_cooler	Air provision
Air provision	ce_air_provision_kiln_system	Air provision
Imbalance aluminium production	al_imbalance	Aluminium produciton imbalance
Scrap provision for aluminium recycling	al_aluminium_recycling_scrap	Provides Aluminium scrap for electrolysis

Process name	Process name (model)	Description
Aluminium dross provision	al_al_dross	Provision of aluminium dross
Water provision for alumina	al_water_provision_alumina	Water provision for alumina production
Provision of diesel fuel	al_diesel_fuel_alumina	Provision of diesel for alumina production
Diesel provision for aluminium	al_diesel_fuel_for_aluminium	Provision of diesel for aluminium production
Mining of iron ore	ext_mining_iron_ore	Mining process extracts iron ore
Mining of limestone	ext_mining_limestone	Mining process extracts limestone
Mining of silica	ext_mining_silica	Mining process extracts silica
Mining of clay	ext_mining_clay	Mining process extracts clay
Mining of gypsum	ext_mining_gypsum	Mining process extracts gypsum
Mining of pozzolana	ext_mining_pozzolana	Mining process extracts pozzolana
Mining of bauxite	ext_bauxite_mining	Mining process extracts mining
Dust from industrial waste	ext_waste_management_dust	Dust from industrial waste
Fly ash from industrial waste	ext_waste_management_fly_ash	Fly ash from industrial waste
Coal extraction	ext_coal_extraction	Coal extraction
Diesel fuel provision	ext_diesel_fuel	Diesel fuel provision
Provision of wood logs	ext_wood_logs	Provision of wood logs
Natural gas extraction	ext_natural_gas_extraction	Natural gas extraction
Aluminium: production of carbon anodes	ext_aluminium_carbon_anodes	Aluminium: production of carbon anodes
Aluminium: production of bath material	ext_aluminium_bath_material	Aluminium: production of bath material
Aluminium: production of argon	ext_aluminium_argon	Aluminium: production of argon
Aluminium: production of alloy additives	ext_alloy_additives	Aluminium: production of alloy additives
Biomass provision for pulping	ext_air_provision	Biomass provision for pulping
Provision of air	ext_provision_combustion_air	Provision of air
Provision of biomass and wood for pulping	ext_biomass_provision_pulping	Provision of biomass and wood for pulping
Provision of chemicals for pulping	ext_chemical_provisions_pulping	Provision of chemicals for pulping
Provision of chemicals (NaOH)	ext_chemicals_provision_NaOH	Provision of chemicals (NaOH)
Provision of chemicals for other processes	ext_chemicals_other	Provision of chemicals for other processes
Provision of other inputs for paper production	ext_paper_other	Provision of other inputs for paper production
Paper collection for recycling	ext_paper_collection_recycling	Paper collection for recycling
Water provision	ext_water_provision	Water provision
Scrap provision for EAF	ext_scrap_eaf_provision	Scrap provision for EAF
Air provision	ext_other_air_provision	Air provision
Slag cooling	hx_slag_cooling1	Slag cooling
Slag cooling	hx_slag_cooling2	Slag cooling
Slag cooling	hx_slag_cooling3	Slag cooling
Slag cooling with heat exchange	hx_slag_cooling1	Cooling slag from steel production with waste heat recovery

Process name	Process name (model)	Description
Slag cooling with heat exchange	hx_slag_cooling2	Cooling slag from steel production with waste heat recovery
Slag cooling with heat exchange	hx_slag_cooling3	Cooling slag from steel production with waste heat recovery
Coking tar HX	hx_coking_tar	Cooling of tar
Sinter flue gas HX	hx_sinter_flue_gas_s11	Sinter flue gas recovery
Coking flue gas HX	hx_blast_stove_steelmaking_s12	Blast stove flue gas recovery
Ironmaking HX	hx_ironmaking_s13	Ironmaking flue gas recovery
Blast stove flue gas HX	hx_blast_stove_steelmaking_s14	Blast stove steelmaking recovery in ORC
Sinter flue gas HX	hx_sinter_flue_gas_s15	Sinter flue gas recovery
BF gas HX	hx_bf_gas_other_s16	Blast furnace gas
Ironmaking HX	hx_ironmaking_s17	Ironmaking flue gas recovery
Coke oven flue gas HX	hx_cooling_coke_oven_s18	Coke oven heat recovery
Coke oven flue gas ORC	hx_cooling_coke_oven_s19	Coke oven heat recovery
Blast furnace gas	hx_bf_gas_other	Blast furnace gas
Clinker cooler flue gas ORC	hx_clinker_cooler_gas_s34	Clinker cooler gas heat recovery
Clinker preheater gas ORC	hx_clinker_preheater_gas_s35	Clinker preheater gas heat recovery
Clinker preheater flue gas HX	hx_clinker_preheater_gas_s36	Clinker preheater gas heat recovery
Aluminium flue gas recovery	hx_aluminium_flue_gas	Aluminium flue gas
Aluminium flue gas recovery	hx_aluminium_flue_gas_s41	Aluminium flue gas heat recovery
Coking tar HX	hx_coking_tar_s61	Coking tar heat exchange
Coal preheating HX	hx_coal_preheating	Coal preheating
Air preheating HX	hx_air_preheating	Air preheating
Flue gas HX/ORC	hx_steam_electricity_ORC	Flue gas heat recovery
Provision of other inputs	other_provision	Other provision of inputs of the production processes

3 Model implementation

This sections provides a description of the model and the implementation in python. Figure S 10 summarises the input files and code used for the industrial symbiosis model. The input files are the material demand, symbiosis scenarios and the production coefficient matrix.

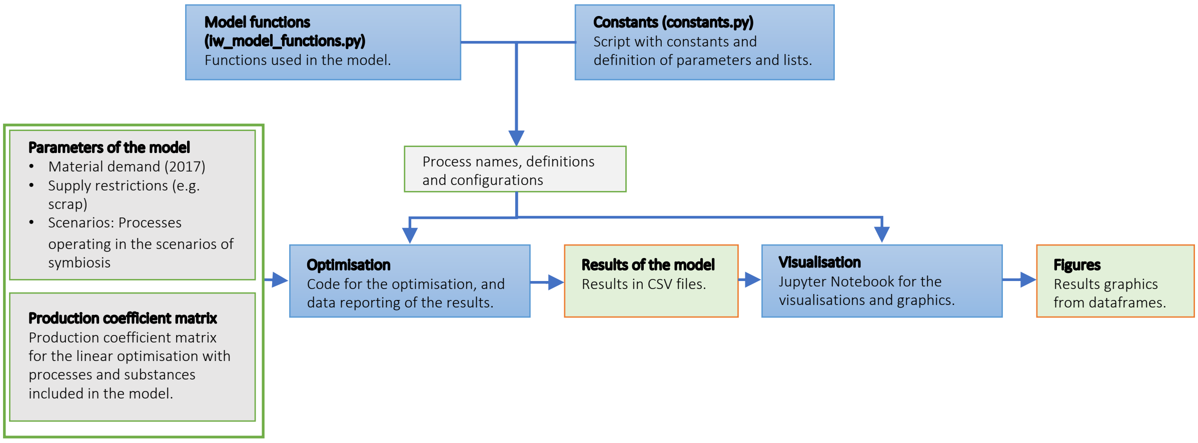

Figure S 10: Overview of the model in python

For the model implementation in python (version 3.9.6), the mathematical programming package scipy (version 1.1.0) is used.

Optimisation of production rates uses the function linear program (linprog) in scipy:
optimise_scenario_ref = linprog(parameters_scenario_ref["ghg"], A_ub=parameters_scenario_ref["A_ub"], b_ub=parameters_scenario_ref["b_ub"], bounds=parameters_scenario_ref["bounds"], method=optimisation_setup["method"], options=optimisation_setup["options"])

4 Sensitivity analysis

The goal of the uncertainty and sensitivity analysis is to assess the robustness of the symbiosis model with regard to changes in the input parameters.

The global symbiosis model has several input parameters which influence the overall system's GHG emissions. The material demand, the production-coefficients, the process-level GHG emissions and the supply constraints are externally defined. Whereas the production coefficient matrix contains the constant production coefficients (recipes), the other three main parameters can be varied to assess the sensitivity and stability of the model results.

- The global material demand ($\hat{y}$),
- The process-specific GHG emissions (g_i),
- The supply constraints, i.e. process rates of the external processes that provide fly ash or secondary resources (c_i),

The sensitivity analysis was conducted (1) for the process-specific GHG emissions and (2) for the constraints on the external supply of fly ash.

Changes in the process-specific GHG emissions

The process-specific GHG emissions (g_i) include the direct process-level GHG emissions of every production process included in the model. The indirect emissions are covered through the energy inputs (e.g. electricity) of the processes with a process-specific GHG emission factor.

The coefficients for the direct emissions for the major bulk material processes were obtained from the literature review. The values (g_i) were varied by ±10% and ±20% for every process. The findings are summarised in Figure S 11 and Figure S 12.

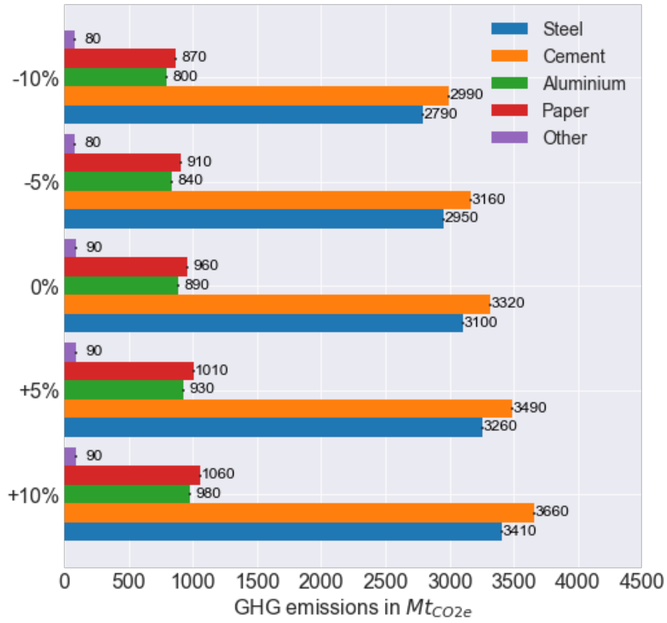

Figure S 11: Results for changes in emission factors of the processes

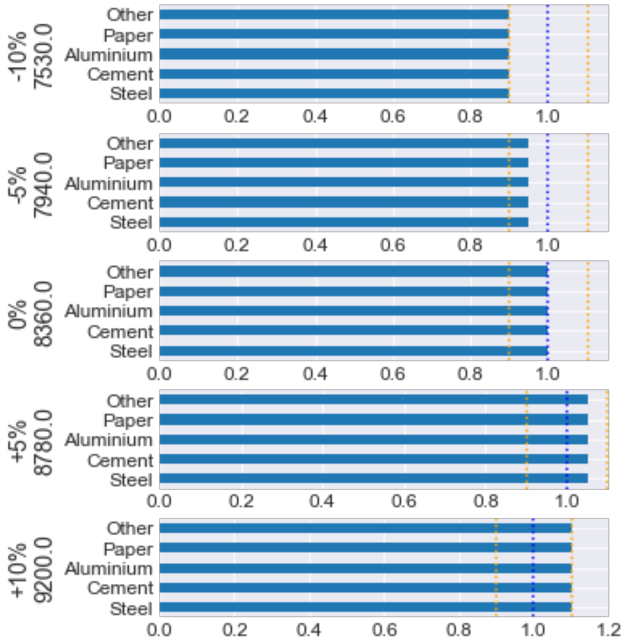

Figure S 12: Results for relative changes in emission factors of the processes

The relative changes of the GHG emissions in the reference scenario are consistent with an increase or decrease of the specific emissions factors. Further research could include a more detailed range and uncertainty reporting of the process-level GHG emissions to explicitly include uncertainty in the GHG emission factors as well as the production coefficients.

Fly ash supply

The emissions reductions in cement production mainly stem from the utilisation of fly ash as a cementitious material (with up to 35% use of fly ash). The cement sector emissions reductions (17%) in scenario A are mainly driven by the utilisation of fly ash. Figure S 13 visualises the impact of increasing fly ash supply on the sectoral GHG emissions in cement production for different levels of fly ash supply.

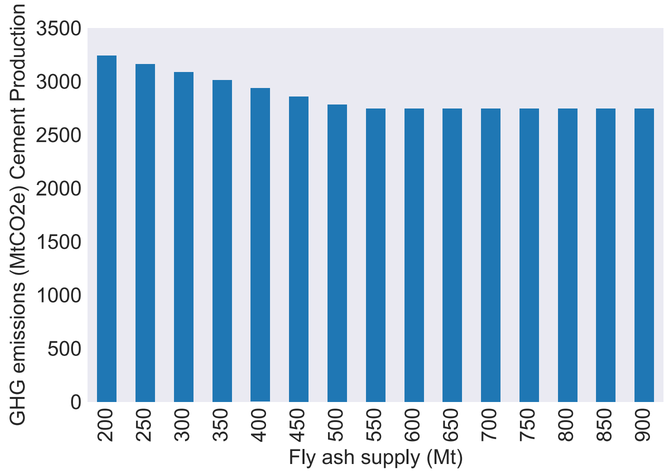

Figure S 13: Sectoral GHG emissions in cement production in scenario A

Overall, the relative changes of overall emissions due to changes of the process-level emission factors lead to a similar increases and reductions in system GHG emissions across all scenarios. The changes in the availability of the secondary materials lead to (small) changes in the overall emissions in the reference scenario. Further research could include a more detailed assessment of changes and uncertainty of the production coefficient matrix.

5 References

(1) Statista. *Cement production global 2019*. https://www.statista.com/statistics/1087115/global-cement-production-volume/ (accessed 2021-06-04).

(2) Statista. *Paper industry*. https://www.statista.com/study/18116/paper-industry--statista-dossier/ (accessed 2021-06-04).

(3) FAO. *Pulp and paper production capacities*. http://www.fao.org/forestry/statistics/80571/en/ (accessed 2021-06-04).

(4) Ritter, S. K. A New Life For Coal Ash. *Chemical & Engineering News*. May 15, 2016.

(5) McBrien, M.; Serrenho, A. C.; Allwood, J. M. Potential for Energy Savings by Heat Recovery in an Integrated Steel Supply Chain. *Applied Thermal Engineering* **2016**, *103*, 592–606. https://doi.org/10.1016/j.applthermaleng.2016.04.099.

(6) Gonzalez Hernandez, A.; Cullen, J.; Paoli, L. Global Average Resource Flow Data for the Steel Industry, 2017. https://doi.org/10.17863/cam.10564.

(7) European Commission. Best Available Techniques (BAT) Reference Document for Iron and Steel Production. 2013.

(8) Carpenter, A. *CO2 Abatement in the Iron and Steel Industry*; IEA Clean Coal Centre, 2012.

(9) World Steel Association. Steel Industry Co-Products: Worldsteel Position Paper, 2018.

(10) Pardo, N.; Moya, J. A. Prospective Scenarios on Energy Efficiency and CO2 Emissions in the European Iron & Steel Industry. *Energy* **2013**, *54*, 113–128. https://doi.org/10.1016/j.energy.2013.03.015.

(11) Krassnig, H.-J.; Luidold, S.; Antrekowitsch, H.; Kleimt, B.; Voj, L. Energie- und Stoffbilanzierung eines 36-t-Elektrolichtbogenofens. *Berg Huettenmaenn Monatsh* **2007**, *152* (9), 287–291. https://doi.org/10.1007/s00501-007-0312-y.

(12) Worrell, E.; Price, L.; Neelis, M.; Galitsky, C.; Zhou, N. *World Best Practice Energy Intensity Values for Selected Industrial Sectors*; LBNL-62806; Ernest Orlando Lawrence Berkeley National Laboratory: Berkeley, CA, USA, 2008.

(13) Gao, T.; Shen, L.; Shen, M.; Liu, L.; Chen, F. Analysis of Material Flow and Consumption in Cement Production Process. *Journal of Cleaner Production* **2016**, *112*, 553–565. https://doi.org/10.1016/j.jclepro.2015.08.054.

(14) Global Cement and Concrete Association. *GNR - GCCA in Numbers*. GCCA. https://gccassociation.org/sustainability-innovation/gnr-gcca-in-numbers/ (accessed 2020-05-31).

(15) Scrivener, K. L.; John, V. M.; Gartner, E. M. *Eco-Efficient Cements: Potential Economically Viable Solutions for a Low-CO2 Cement- Based Materials Industry*; Report; United Nations Environment Program, 2016.

(16) Huntzinger, D. N.; Eatmon, T. D. A Life-Cycle Assessment of Portland Cement Manufacturing: Comparing the Traditional Process with Alternative Technologies. *Journal of Cleaner Production* **2009**, *17* (7), 668–675. https://doi.org/10.1016/j.jclepro.2008.04.007.

(17) Holcim; GTZ. *Guidelines on Co-Processing Waste Materials in Cement Production The GTZ-Holcim Public Private Partnership*; The GTZ-Holcim Public Private Parternship, GTZ and Holcim Group Support Ltd.: Germany, 2006.

(18) Karellas, S.; Leontaritis, A.-D.; Panousis, G.; Bellos, E.; Kakaras, E. Energetic and Exergetic Analysis of Waste Heat Recovery Systems in the Cement Industry. *Energy* **2013**, *58*, 147–156. https://doi.org/10.1016/j.energy.2013.03.097.

(19) Fellaou, S.; Bounahmidi, T. Analyzing Thermodynamic Improvement Potential of a Selected Cement Manufacturing Process: Advanced Exergy Analysis. *Energy* **2018**, *154*, 190–200. https://doi.org/10.1016/j.energy.2018.04.121.

(20) Balomenos, E.; Davris, P.; Pontikes, Y.; Panias, D. Mud2Metal: Lessons Learned on the Path for Complete Utilization of Bauxite Residue Through Industrial Symbiosis. *Journal*

of Sustainable Metallurgy **2017**, *3* (3), 551–560. https://doi.org/10.1007/s40831-016-0110-4.
(21) Nowicki, C.; Gosselin, L. An Overview of Opportunities for Waste Heat Recovery and Thermal Integration in the Primary Aluminum Industry. *JOM* **2012**, *64* (8), 990–996. https://doi.org/10.1007/s11837-012-0367-4.
(22) European Commission; Cusano, G.; Gonzalo, M.; Farrell, F.; Remus, R.; Roudier, S.; Sancho, L. D. *Best Available Techniques (BAT) Reference Document for the Non-Ferrous Metals Industries*; JRC107041; European Commission: Luxembourg, 2017.
(23) World Aluminium. World Aluminium — Primary Aluminium Production, 2020.
(24) World Aluminium. *TECHNOLOGY ROADMAP MAXIMISING THE USE OF BAUXITE RESIDUE IN CEMENT*; 2020.
(25) Barzi, Y. M.; Assadi, M.; Parham, K. A Waste Heat Recovery System Development and Analysis Using ORC for the Energy Efficiency Improvement in Aluminium Electrolysis Cells. *Int J Energy Res* **2018**, *42* (4), 1511–1523. https://doi.org/10.1002/er.3940.
(26) Corcelli, F.; Fiorentino, G.; Vehmas, J.; Ulgiati, S. Energy Efficiency and Environmental Assessment of Papermaking from Chemical Pulp - A Finland Case Study. *Journal of Cleaner Production* **2018**, *198*, 96–111. https://doi.org/10.1016/j.jclepro.2018.07.018.
(27) Christensen, T. H.; Damgaard, A. Recycling of Paper and Cardboard. In *Solid Waste Technology & Management*; John Wiley & Sons, Ltd, 2010; pp 201–210. https://doi.org/10.1002/9780470666883.ch15.
(28) European Commission. Best Available Techniques (BAT) Reference Document for the Production of Pulp, Paper and Board. 2015.
(29) Ecoinvent. Pulp, 2020.
(30) Öko-Institut. Prozessdetails: Papier-PappeKraftpapier Gebleicht, 2011.
(31) Rogers, J. G.; Cooper, S. J.; Norman, J. B. Uses of Industrial Energy Benchmarking with Reference to the Pulp and Paper Industries. *Renewable and Sustainable Energy Reviews* **2018**, *95*, 23–37. https://doi.org/10.1016/j.rser.2018.06.019.
(32) Van Ewijk, S.; Stegemann, J. A.; Ekins, P. Global Life Cycle Paper Flows, Recycling Metrics, and Material Efficiency. *Journal of Industrial Ecology* **2017**, *22* (4), 686–693. https://doi.org/10.1111/jiec.12613.
(33) World Aluminium. LIFE CYCLE INVENTORY DATA AND ENVIRONMENTAL METRICS FOR THE PRIMARY ALUMINIUM INDUSTRY: 2015 Data, 2017.
(34) World Aluminium. *World Aluminium — Primary Aluminium Smelting Energy Intensity*. https://www.world-aluminium.org/statistics/primary-aluminium-smelting-energy-intensity/ (accessed 2021-04-14).
(35) Balomenos, E.; Panias, D.; Paspaliaris, I. Energy and Exergy Analysis of the Primary Aluminum Production Processes: A Review on Current and Future Sustainability. *Mineral Processing and Extractive Metallurgy Review* **2011**, *32* (2), 69–89. https://doi.org/10.1080/08827508.2010.530721.
(36) International Energy Agency. Global Energy & CO2 Status Report: The Latest Trends in Energy and Emissions in 2018. *IEA* **2019**.
(37) BEIS. Greenhouse Gas Reporting: Conversion Factors 2019, 2019.
(38) Worrell, E.; Blinde, P.; Neelis, M.; Blomen, E.; Masanet, E. *Energy Efficiency Improvement and Cost Saving Opportunities for the U.S. Iron and Steel Industry An ENERGY STAR(R) Guide for Energy and Plant Managers*; LBNL-4779E; Lawrence Berkeley National Lab. (LBNL), Berkeley, CA (United States), 2010. https://doi.org/10.2172/1026806.
(39) GHG Protocol. Emission Factors from Cross-Sector Tools, 2017.
(40) Smil, V. Chapter 7 - Energy Costs and Environmental Impacts of Iron and Steel Production: Fuels, Electricity, Atmospheric Emissions, and Waste Streams. In *Still the Iron Age*; Smil, V., Ed.; Butterworth-Heinemann: Boston, 2016; pp 139–161. https://doi.org/10.1016/B978-0-12-804233-5.00007-5.
(41) Paoli, L.; Cullen, J. Technical Limits for Energy Conversion Efficiency. *Energy* **2020**, *192*, 116228. https://doi.org/10.1016/j.energy.2019.116228.

(42) Rogers, G. F. C.; Mayhew, Y. R. *Thermodynamic and Transport Properties of Fluids: SI Units*, 5. ed., reprinted; SI units.; Blackwell: Oxford, 2003.
(43) Fakheri, A. Efficiency Analysis of Heat Exchangers and Heat Exchanger Networks. *International Journal of Heat and Mass Transfer* **2014**, *76*, 99–104. https://doi.org/10.1016/j.ijheatmasstransfer.2014.04.027.
(44) World Steel Association. *Steel Industry By-Products*; World Steel Association, 2010.
(45) Lecompte, S.; Oyewunmi, O. A.; Markides, C. N.; Lazova, M.; Kaya, A.; Van den Broek, M.; De Paepe, M. Case Study of an Organic Rankine Cycle (ORC) for Waste Heat Recovery from an Electric Arc Furnace (EAF). *Energies* **2017**, *10* (5), 649. https://doi.org/10.3390/en10050649.
(46) Ramirez, M.; Epelde, M.; de Arteche, M. G.; Panizza, A.; Hammerschmid, A.; Baresi, M.; Monti, N. Performance Evaluation of an ORC Unit Integrated to a Waste Heat Recovery System in a Steel Mill. *Energy Procedia* **2017**, *129*, 535–542. https://doi.org/10.1016/j.egypro.2017.09.183.
(47) Element Energy; Ecofys; Imperial College; Paul Stevenson; Robert Hyde. *The Potential for Recovering and Using Surplus Heat from Industry*; 2014.
(48) Pöyry Energy Consulting. The Potential and Costs of District Heating Networks: A Report to the Department of Energy and Climate Change, 2009.
(49) Atienza-Márquez, A.; Bruno, J. C.; Coronas, A. Recovery and Transport of Industrial Waste Heat for Their Use in Urban District Heating and Cooling Networks Using Absorption Systems. *Applied Sciences* **2020**, *10* (1), 291. https://doi.org/10.3390/app10010291.
(50) Santin, M.; Chinese, D.; De Angelis, A.; Biberacher, M. Feasibility Limits of Using Low-Grade Industrial Waste Heat in Symbiotic District Heating and Cooling Networks. *Clean Techn Environ Policy* **2020**, *22* (6), 1339–1357. https://doi.org/10.1007/s10098-020-01875-2.
(51) Ammar, Y.; Joyce, S.; Norman, R.; Wang, Y.; Roskilly, A. P. Low Grade Thermal Energy Sources and Uses from the Process Industry in the UK. *Applied Energy* **2012**, *89* (1), 3–20. https://doi.org/10.1016/j.apenergy.2011.06.003.
(52) Palmer, J.; Livingstone, M.; Adams, A. WHAT DOES IT COST TO RETROFIT HOMES? - Updating the Cost Assumptions for BEIS's Energy Efficiency Modelling, 2017.
(53) Bürger, V.; Steinbach, J.; Kranzl, L.; Müller, A. Third Party Access to District Heating Systems - Challenges for the Practical Implementation. *Energy Policy* **2019**, *132*, 881–892. https://doi.org/10.1016/j.enpol.2019.06.050.
(54) Korpela, T.; Kaivosoja, J.; Majanne, Y.; Laakkonen, L.; Nurmoranta, M.; Vilkko, M. Utilization of District Heating Networks to Provide Flexibility in CHP Production. *Energy Procedia* **2017**, *116*, 310–319. https://doi.org/10.1016/j.egypro.2017.05.077.
(55) Vandermeulen, A.; van der Heijde, B.; Helsen, L. Controlling District Heating and Cooling Networks to Unlock Flexibility: A Review. *Energy* **2018**, *151*, 103–115. https://doi.org/10.1016/j.energy.2018.03.034.
(56) Pili, R.; García Martínez, L.; Wieland, C.; Spliethoff, H. Techno-Economic Potential of Waste Heat Recovery from German Energy-Intensive Industry with Organic Rankine Cycle Technology. *Renewable and Sustainable Energy Reviews* **2020**, *134*, 110324. https://doi.org/10.1016/j.rser.2020.110324.
(57) Hashimoto, S.; Fujita, T.; Geng, Y.; Nagasawa, E. Realizing CO2 Emission Reduction through Industrial Symbiosis: A Cement Production Case Study for Kawasaki. *Resources, Conservation and Recycling* **2010**, *54* (10), 704–710. https://doi.org/10.1016/j.resconrec.2009.11.013.
(58) Hinkel, M.; Blume, S.; Hinchliffe, D.; Mutz, D.; Hengevoss, D. Guidelines on Pre-and Co-Processing of Waste in Cement Production. *Gesellschaft Fur Internationale Zusammenarbeit GmbH (GIZ)* **2020**.
(59) Bhatia, P.; Cummis, C.; Draucker, L.; Rich, D.; Lahd, H.; Brown (WBCSD), A. Greenhouse Gas Protocol Product Life Cycle Accounting and Reporting Standard. **2011**.

(60) Pontikes, Y.; Angelopoulos, G. N. Bauxite Residue in Cement and Cementitious Applications: Current Status and a Possible Way Forward. *Resources, Conservation and Recycling* **2013**, *73*, 53–63. https://doi.org/10.1016/j.resconrec.2013.01.005.
